# Selection of Autochthonous Yeasts Isolated from the Intestinal Tracts of Cobia Fish (*Rachycentron canadum)* with Probiotic Potential

**DOI:** 10.3390/jof9020274

**Published:** 2023-02-18

**Authors:** Samira Reinoso, María Soledad Gutiérrez, Cristóbal Domínguez-Borbor, Wilfrido Argüello-Guevara, Milton Bohórquez-Cruz, Stanislaus Sonnenholzner, Daniela Nova-Baza, Claudia Mardones, Paola Navarrete

**Affiliations:** 1 Microbiology and Probiotics Laboratory, Institute of Nutrition and Food Technology (INTA), University of Chile, Avenida El Líbano 5524, Macul, Santiago 7830490, Chile; 2 ESPOL Polytechnic University, Escuela Superior Politécnica del Litoral, ESPOL, Centro Nacional de Acuicultura e Investigaciones Marinas, CENAIM, Guayaquil 090211, Ecuador; 3 ESPOL Polytechnic University, Escuela Superior Politécnica del Litoral, ESPOL, Facultad de Ingeniería Marítima y Ciencias del Mar, FIMCM, Guayaquil 090211, Ecuador; 4 Departamento de Análisis Instrumental, Facultad de Farmacia, Universidad de Concepción, Concepción 4070386, Chile

**Keywords:** yeast, probiotic, cobia, *Rachycentron canadum*, *Debaryomyces hansenii*, *Candida haemuloni*, fish larvae

## Abstract

Some yeast strains have been proposed as probiotics to improve the health of cultured fish. Cobia is a tropical benthopelagic fish species with potential for marine aquaculture; however, one of the main limitations to its large-scale production is the high mortality of fish larvae. In this study, we evaluated the probiotic potential of autochthonous yeasts from the intestines of cobia. Thirty-nine yeast isolates were recovered from the intestinal mucosa of 37 adult healthy cobia by culture methods. Yeasts were identified by sequencing of the ITS and D1/D2 regions of the 28S rRNA gene and typed by RAPD-PCR using the M13 primer. Yeast strains with unique RAPD patterns were characterized in terms of their cell biomass production ability; anti-*Vibrio*, enzymatic, and hemolytic activity; biofilm production; hydrophobicity; autoaggregation; polyamine production; safety; and protection of cobia larvae against saline stress. *Candida haemuloni* C27 and *Debaryomyces hansenii* C10 and C28 were selected as potential probiotics. They did not affect the survival of larvae and showed biomass production >1 g L^−1^, hydrophobicity >41.47%, hemolytic activity γ, and activity in more than 8 hydrolytic enzymes. The results suggest that the selected yeast strains could be considered as potential probiotic candidates and should be evaluated in cobia larvae.

## 1. Introduction

In the last thirty years, aquaculture production has significantly increased worldwide, and marine fish aquaculture has become a successful activity in several countries [1]. This high level of production has been linked to several decades of scientific research and the investment of significant economic resources. However, the sector is still facing several challenges in relation to the optimization of the production processes and the reduction of the impacts of different bottlenecks, such as the high mortality rate of individuals in the early stage of fish development [2].

Cobia (*Rachycentron canadum*) is a tropical benthopelagic species that is widely distributed in tropical and subtropical marine waters worldwide, except for in the Eastern Pacific [3]. Cobia farming has been reported since the 1990s in Taiwan, and its juvenile production technologies have been described since 1997 [4]. Its economic potential is associated with its good meat quality, rapid growth, and easy adaptability to captivity [5,6] and its high demand among Asian consumers, especially in Taiwan and Japan [7]. The increase in demand for cobia is evident worldwide, is its production expanded from 3 T in 1995, to 40,522 T in 2020 [8]. Similarly, a growing catch rate has been reported, from 25 T in 1950 to 15,173 T in 2020 [8].

The supply of high-quality juveniles for large-scale production is one of the main limitations to intensifying the production of marine species [9], including cobia [6,10,11,12]. Therefore, during the last 30 years, scientific research has been focused on the study of the morphophysiological development of larvae and juveniles of various fish species, especially of the digestive tract and its functionality [13] to promote healthy nutrition [9]. In cobia, the use of bacterial probiotics [14,15], weaning strategies [10,11], culture systems [16,17,18], dietary supplementation with taurine [19,20], and commercial mannan oligosaccharides [21] have been evaluated to increase larval development and juvenile production.

The use of probiotics is a strategy to improve fish performance and has been used in all development stages [22]. From an aquaculture perspective, a probiotic is a living microbial complement that has a beneficial effect on the host by modifying its associated microbial community with the purpose being to guarantee better use of the food or increasing its nutritional value, improving the response of the host to a disease, and improving the quality of their environment [23].

In the aquaculture industry, most of the microorganisms used as probiotics are bacteria [24]; however, an increased interest in yeasts has been described in the last years [25]. Yeasts are usual inhabitants of the marine environment [25] and are commensal organisms of the fish intestine [26]. They show a low pathogenic potential, and they can contribute several benefits to fish health [27]. They are naturally resistant to antibacterial compounds [28,29], which are frequently used in the aquaculture sector [30]. When they are administered orally, yeasts can adhere to the fish mucus [31,32,33] and survive in the gastrointestinal tract [34], which could favor a prolonged probiotic effect. Additionally, in vitro studies have shown that yeasts can grow on the intestinal mucosa [31,32], which can also favor their survival and persistence in the host gut.

The potential of yeasts to be used as probiotics is mainly based on their ability to produce several compounds of high biological and nutritional value, such as proteins, lipids, vitamins, pigments, nucleic acids [35], enzymes [26,36], and polyamines [37,38]. In addition, some compounds of their cell walls, such as β-glucans and mannoproteins, stimulate the innate immune and antioxidant systems of the fish [27,39] and protect them from bacterial [34,40,41,42] and viral [41] infections.

Yeasts have been identified in healthy marine fish [26] with a prevalence of cultivable yeasts ranging from 66% in the early stages [43] to 74.8% [26] to 100% in adult fish [43]. The concentration of yeasts tends to be highly variable between species, and it is possible to find up to 7 log_10_ colony-forming units (CFU) g^−1^ of intestinal content [27]. Although they often represent less than 1% of the total microbial isolates, they can be incorporated at low concentrations (4 log_10_ CFU g^−1^ on a body weight basis [44]) and generate beneficial effects on the host, since they have cell volumes that can be one-hundred-fold greater than that of bacteria [27]. To date, no study has investigated the cultivable intestinal mycobiota of cobia and their potential probiotic effect on this host. Due to the increasing economic significance of this fish species, we isolated, identified, and selected potential probiotic strains based on their in vitro benefic properties.

## 2. Materials and Methods

### 2.1. Fish and Intestinal Samples

A total of 37 healthy adult cobia weighing 236.2–5360.0 g were collected from the National Aquaculture and Marine Research Center (CENAIM-ESPOL) (n= 31), located in San Pedro, Santa Elena, and from the company Emagrocom S.A. (n= 6), located in Jaramijó, Manabí, Ecuador. The health status was confirmed by the visual inspection of fish. Fish with normal swimming and feeding behaviors and no signs of bacterial or viral diseases were considered healthy. Fish were fed with two diets, formulated feed (FF) (n= 13; Gisis S.A of Skretting; 7 mm, 12% moisture, 40% protein, 31% carbohydrates, and 8% lipids) and frozen fish pieces (FFP) (n = 24; mixture of local fish: *Auxis* sp., *Scomber japonicus,* and *Opisthonema* sp., 73.5% moisture, 18.1% protein, and 5.8% lipids [45]). At the CENAIM-ESPOL, fish were reared under laboratory conditions in an open-flow system with a salinity level of 35 g L^−1^, a natural photoperiod (12: 12 LD), and a temperature of 26.60 ± 0.72 °C, while at Emagrocom S.A., fish were reared under laboratory conditions in an open-flow system with a salinity level of 29 g L^−1^, a natural photoperiod (12: 12 LD), and a temperature of 27.02 ± 1.40 °C. Fish were sampled in February 2021. For the intestinal sampling, fish were fasted for 24 h prior to sacrifice with an overdose of anesthesia consisting of fish immersion in a water solution containing 50 mg L^−1^ eugenol (Eufar S.A., Bogotá, Colombia). The entire intestine of each fish was removed under sterile conditions, dissected with sterile scissors, and washed with sterile saline solution (NaCl, 0.89%). The intestinal epithelial layer was scraped with a sterile scalpel, and the scraped mucosa was gently transferred to sterile 1.5 mL tubes.

### 2.2. Isolation of Yeast from Intestinal Samples by Culture Method

The isolation process was performed in accordance with [26,36] with slight modifications. The mucosa was weighed (approximately 250 mg) and homogenized with a micropestle in 10 volumes of sterile phosphate-buffered saline (PBS). Three tenfold serial dilutions were made with sterile PBS. One hundred microliters of each dilution was plated, in duplicate, on yeast-extract peptone dextrose medium (YPD; 1% yeast extract; 2% peptone; 2% glucose, and 2% agar) supplemented with 0.05% chloramphenicol. Plates were incubated at 30 °C for up to seven days, and all colonies with different morphologies from plates containing 1–100 colonies were purified by transferring each colony to new plates. Each isolate and purified colony was microscopically confirmed to be yeast according to its cell morphology (Olympus CX31, Lifescience, Tokyo, Japan). All isolates were cultured in YPD medium supplemented with agar (2%) for 24 h. Then, the colonies were resuspended in YPD broth with 15% glycerol (v/v) as a cryopreservative agent and stored at −80 °C for further analysis. 

### 2.3. DNA Extraction

Genomic DNA from each yeast isolate was extracted using glass beads (425–600 µm, Sigma, St Louis, MO, USA) and a phenol-chloroform solution, as described previously [46], with slight modifications. Briefly, 250 µL of each isolate grown in YPD broth was centrifuged (DLAB Scientific D3024R, Beijing, China), and the supernatant was removed. Then, 600 µL of TE buffer (25 mM Tris HCl, 10 mM EDTA, pH 8), 200 mg of glass beads) and 600 µL of phenol: chloroform: isoamyl alcohol (25:24:1) were added. This mixture was vortexed at maximum speed for 10 min and centrifuged at 16,000× *g* for 5 min. The supernatant (600 µL) was transferred to a 1.5 mL tube, and 600 µL of chloroform: isoamyl alcohol (24:1) was added, vortexed for 30 s, and centrifuged at 16,000× *g* for 1 min. The aqueous phase (400 µL) was transferred to a 1.5 mL tube, 800 µL of cold absolute ethanol was added to precipitate the DNA, and the tube was kept at −20 °C for 2 h. The tube was then centrifuged at 18,000× *g* for 10 min, the ethanol was removed, and the pellet was resuspended in 300 µL of 70% ethanol. The tube was centrifuged at 18,000× *g* for 2 min. The ethanol was removed, and the pellet was dried on an oven at 45 °C for 45 min. Finally, the pellet was resuspended in 50 µL of TE-buffer containing RNaseA (20 mg mL^−1^) and stored at −20 °C until use. The DNA quantity and quality were determined by spectrophotometry (Eppendorf Biophotometer 6131, Hamburg, Germany) and agarose gel electrophoresis (1.5%), respectively.

### 2.4. Sequencing for Yeast Identification

For taxonomic identification, DNA from each yeast isolate was sent to Macrogen (Seoul, Republic of Korea) for amplification, purification, and sequencing of the ITS1, 5.8S, ITS2, and D1/D2 regions of the 28S rRNA gene. The forward primers were ITS1 (5′-TCCGTAGGTGAACCTGCGG-3′) or ITS5 (5′-GGAAGTAAAAGTCGTAACAAGG-3′) and reverse primers were NL4 (5′-GGTCCGTGTTTCAAGACG-3′) or ITS4 (5′-TCCTCCGCTTATTGATATGC-3′). Electropherograms were edited using Geneious Prime 2021.2.2 software, and the edited sequences were compared with sequences in the NCBI and checked in the Mycobank database. All sequences were submitted to the NCBI database under accession numbers OQ184038–OQ184076.

### 2.5. Specific PCR for Debaryomyces hansenii Identification

Yeast isolates identified as *Debaryomyces* spp. were identified as *D. hansenii* using the species-specific primers reported by [47]. These primers amplified a putative PAD1 gene homologous region (729 bp). PCR was performed on DNA extracted from the yeast isolates using the primers DhPadF 5′ GCGACTATGAACAGGTTTCCAACGA 3′ and DhPadR 5′CCTTCAATGTAACATCAGCGGCCC 3′. The amplification reaction was performed in a total volume of 10 µL containing 1 µL of yeast DNA (100 ng µL^−1^), 2 µL of nuclease-free water, 5 µL of GoTaq Master Mix 2× (Promega Corporation, USA), and 1 µL of each primer (10 µM). The thermal cycler (TProfessional Basic Gradient, Biometra Analytic Jena GmbH, Germany) was operated under the following amplification conditions: initial denaturation at 94 °C for 5 min, followed by 39 cycles consisting of 30 s at 94 °C, 30 s at 55 °C, 1 min at 72 °C, and a final extension of 10 min at 72 °C. PCR products were separated on a 1% agarose gel using 1× TAE buffer at 80 V for 30 min. The size of the amplification products was determined by comparison with a 100 bp DNA ladder (Invitrogen™, Thermo Scientific™, Waltham, MA, USA).

### 2.6. RAPD-PCR Profile

Yeast isolates were analyzed by RAPD-PCR using the M13 primer (5′-GAGGGTGGCGGTTCT-3′), as previously described [48,49]. The amplification reaction was performed in a total volume of 10 µL containing 100 ng of DNA (2 µL), 20 mM Tris-HCl (1 µL), 0.2 mM of dNTPs (0.2 µL), 4.45 mM MgCl_2_ (0.89 µL), 1 U of Taq polymerase (0.1 µL), nuclease-free water (3.31 µL), and 2.5 µM of M13 primer (2.5 µL). The thermal cycler (Applied Biosystems™ Veriti™ 96-well Thermal Cycler, USA) was operated under the following amplification conditions: initial denaturation at 94 °C for 4 min, followed by 39 cycles consisting of 5 s at 94 °C, 45 s at 46 °C, 1 min at 72 °C, and a final extension of 5 min at 72 °C. PCR products were separated on 2% agarose gel using 1× TAE buffer at 90 V for 2 h. Gels were stained with SYBr SAFE 2000× (Invitrogen™, Thermo Scientific™, USA), and the size of the amplification products was determined by comparison with a 100 bp DNA ladder (Invitrogen™, Thermo Scientific™, USA) using GelAnalyzer 19.1 software. Dendrograms were constructed using PAST software with the UPGMA method and Jaccard distance. Isolates with more than 85% similarity were assigned to the same strain. 

### 2.7. Yeast Biomass Production

Yeast isolates were cultured in 5 mL of YPD broth at 28 °C with continuous agitation (150 rpm) for 40 h. The yeasts were then centrifuged at 10,000× *g* for 5 min, the supernatant was removed, and the biomass was dried in an oven at 100 °C for 12 h. The dried biomass was weighed on an analytical balance. 

### 2.8. Enzymatic Activity

The enzymatic activity of the yeast strains was determined using the API ZYM test (BioMerieux, Lyon, France) in accordance with the manufacturer’s instructions. This test is a semiquantitative method that detects 19 enzymes, including proteases, glycosidases, lipases, and phosphatases. The concentrations of the yeast suspensions were adjusted with a standard 0.5 McFarland barium sulfate solution, which simulates a concentration of 1 × 10^6^ CFU mL^−1^ of yeast approximately [50,51]. After 48 h of culture in YPD broth, yeasts were recovered from the culture medium by centrifugation (10,000× *g* for 5 min), resuspended in sterile PBS, and adjusted to an optical density of 5 McFarland. Then, 65 µL of this suspension was deposited in the 20 wells of each gallery. The galleries were placed in an incubation chamber and incubated at 37 °C for 5 h. Enzyme activity was recorded as positive (+), medium (±), or negative (−) according to the color intensity of each reaction compared with an API-ZYM color reaction chart [36]. Medium and positive enzyme activity levels were considered positive.

### 2.9. Hemolytic Activity

The hemolytic activity of yeast strains was tested using TSA (tryptic soy agar) supplemented with 5% of cobia blood and 0.05% chloramphenicol. To obtain cobia blood, 3 healthy adults were sedated (25 mg L^−1^ eugenol), and 5 mL of blood was collected from each one. The blood was mixed and defibrinated by constant agitation with glass beads (250 µm) for 10 min. Then, 12 mL of blood was mixed with 228 mL of TSA containing 0.05% chloramphenicol. Yeasts were inoculated in cobia blood TSA for 48 h, and hemolytic activity was observed. Yeasts producing α or β hemolysis were considered hemolytic, and γ hemolysis was considered nonhemolytic [52].

### 2.10. Antagonistic Activity against Vibrio spp

We evaluated the antagonistic activity of yeast strains against 5 pathogenic strains of *Vibrio* spp.: *Vibrio harveyi* E22, *Vibrio campbellii* LM2013, *Vibrio parahemolyticus* BA94C2, *Vibrio vulnificus* S2, previously evaluated in [53,54], and *Vibrio anguillarum* PF4, previously evaluated in [34,55]. Antagonistic activity was tested using the agar-well diffusion method described in [56] with slight modifications. Briefly, a 7 h culture of each *Vibrio* in TSB (Tryptic Soy Broth, BD Difco™) was adjusted to 1 × 10^5^ cell mL^−1^, and 50 µL was plated over the entire surface of a TSA agar (tryptic soy agar, BD Difco™) using a sterile hyssop. The plates were then dried, and circular holes were cut in the agar. Each yeast was cultured in YPD broth at 30 °C and 150 rpm for 48 h. One hundred microliters of this culture and 0.2 µm of its filtered supernatant were inoculated into triplicate holes for each pathogen. One hundred microliters of 50 ppm of florfenicol and 50 ppm of chloramphenicol were used as positive controls. Plates were incubated at 30 °C, and the inhibition halo was measured after 24 h. An inhibition halo of greater than 1 mm in diameter was considered positive.

### 2.11. Biofilm Production 

The biofilm production of yeast strains was determined by crystal violet staining, as previously described [57]. A 48-h yeast culture in YPD broth was adjusted to 0.5 McFarland (~1 × 10^6^ CFU mL^−1^). Then, 20 µL of each yeast was inoculated into 4 wells of a sterile 96-well plate containing 180 µL of YPD broth. After 48 h of incubation at 30 °C, the medium was carefully removed, and the plates were dried for 1 h at 45 °C. The biofilms in the wells were then stained with 200 μL of crystal violet (0.1%, *w*/*v*) for 15 min, washed with abundant water, and the insolubilized dye was solubilized by adding 200 μL of ethanol and homogenized. The absorbance at 600 nm was measured with a microplate reader (Varioskan™ LUX, Thermo Scientific) and using ethanol as a blank. If a yeast showed an absorbance > 0.8, it was considered a biofilm producer.

### 2.12. Hydrophobicity Test

Yeast hydrophobicity was determined by measuring the microbial adhesion to organic solvents in accordance with [58] with slight modifications. We used xylene (nonpolar solvent), chloroform (monopolar and acidic solvent), and ethyl acetate (monopolar and basic solvent). Yeast adhesion to xylene reflects the hydrophobicity or hydrophilicity of the cell surface. Adhesion to chloroform and ethyl acetate was considered a measure of electron-donating (basic) and electron-accepting (acidic) yeasts, respectively [59]. Briefly, yeasts grown for 48 h in YPD broth were collected by centrifugation at 10,000× *g* for 5 min, washed twice with PBS, and resuspended in 500 µL PBS. Then, absorbance at 600 nm (A_0_) was measured. The cell suspension was then mixed with equal volumes of each solvent by vortexing for 5 min and then holding them at room temperature for 30 min. The absorbance in the aqueous phase was measured at 600 nm (A_1_). The percentage of hydrophobicity (H%) was calculated using the formula H% = 1 − A_1_/A_0 ×_ 100. Each assay was performed in duplicate. According to the criterion of [60], the hydrophobicity was considered high if the H% in xylene was greater than 80%, medium if the H% was 20–80%, and low if the H% was less than 20%. Medium and high H% (>20%) were considered positive. The H% in ethyl acetate and chloroform was determined to identify the ability of yeast strains to accept or donate electrons, respectively.

### 2.13. Autoaggregation Test

The autoaggregation of yeast strains was performed according to the method described by [52] with some modifications. Four milliliter yeast suspensions were adjusted to an absorbance of 1 at 600 nm in PBS in borosilicate tubes (12 × 75 mm, Schott, Zwiesel, Germany) in triplicate. After vortexing for at least 10 s, all suspensions were kept at room temperature. An aliquot of the upper suspension (200 µL) was carefully collected from each tube, and the absorbance (A) was measured at 600 nm at 0, 1, and 24 h. The percentage of autoaggregation was calculated according to the following equation: %autoaggregation = [1 − A1/A0] × 100, where A0 is the A600 of the yeast suspension at time 0 and A1 represents A600 of the yeast suspension at different times (1 and 24 h). An autoaggregation of >20% was considered positive.

### 2.14. Polyamine Production

Polyamine production by yeast strains was quantified in the supernatants and cell pellets obtained from yeast cultured in triplicate in Synthetic Dextrose Minimal (SDM) broth (2% glucose, 0.67% yeast nitrogen base without amino acids) at 30 °C and 150 rpm for 48 h. SDM broth was used to grow yeasts instead of YPD, which contains polyamines from yeast extract. Two 1 mL aliquots of yeast cultured in SDM were centrifuged at 10,000× *g* for 5 min, and the supernatant was filtered through 0.2 µm. Sterile supernatants and yeast pellets were stored at −20 °C until polyamine analysis. Polyamine quantification was performed in a UHPLC-RF (Nexera LC-30AD, Shimadzu, Kyoto, Japan) equipped with a Kinetex C18 column, a pore size of 100 Å, a length of 100 mm, an internal diameter of 4.6 mm, and a particle size of 2.6 mm. Sample and standard derivatization was performed as previously described [37,61]. The detection of derivatized polyamines with dansyl chloride (Supelco-03641, Sigma, St Louis, MO, USA) was performed by fluorescence using 330 and 520 nm as excitation and emission wavelengths, respectively. Dansylated putrescine (P-7505, Sigma, St Louis, MO, USA), spermidine (S-0381, Sigma, St Louis, MO, USA), and spermine (S-2876, Sigma, St Louis, MO, USA) were used to construct the standard curves. Polyamine concentrations were expressed as ng of each polyamine per dry weight of yeast (ng mg^−1^).

### 2.15. Effect of Yeast Strains on Larval Survival

The in vivo safety of yeast strains was evaluated in cobia larvae at 2 dph (days post-hatching). At this stage, all larvae have opened their mouths [62]. Each yeast was tested in a beaker (100 larvae per beaker) containing 500 mL of seawater in triplicate. Larvae were fed with 3 rotifers (*Brachionus rotundiformis*) mL^−1^ and maintained at 26 °C with continuous aeration. Flasks were inoculated by immersion with 10^7^ CFU mL^−1^ of each yeast (wet biomass) in triplicate. A control group without the addition of yeasts was included. Larval mortality was recorded every hour for 24 h. To confirm yeast consumption by larvae, one of the bottles was inoculated with stained yeasts (with 0.1% acridine orange for 10 min). One hour after inoculation, the larvae were observed under an epifluorescence microscope (Nikon Eclipse E200, Tokyo, Japan).

### 2.16. Protective Effect of Cobia Larvae by Yeasts against a Salinity Stress

At 4 dph, larvae were distributed (in triplicate) in 24 beakers containing 800 mL of seawater (100 larvae per beaker) at 35 g L^−1^ salinity and fed with rotifers enriched with each yeast strain. A control group of larvae was fed rotifers that were not enriched with yeast strains. At 6 dph, 10 larvae from each bottle were exposed to salinity stress by transferring larvae to Petri dishes containing water at 26 °C and 5 g L^−1^ salinity, as described in [63] with modifications. Larval survival was recorded every hour for 8 h.

### 2.17. Yeast Growth Curve

The growth curve was constructed to characterize the 3 selected yeast strains as potential probiotics. Yeasts were inoculated at 1 × 10^5^ cells mL^−1^ (adjusted by optical density) in YPD broth and incubated for 48 h at 28 °C and 150 rpm. Absorbance was measured at 600 nm every 6 h using a microplate reader. 

### 2.18. Viability of Yeast Cells Stored at 4 °C

This assay was performed to evaluate the 3 yeast strains selected as potential probiotics. Yeasts were cultured in YPD broth at 28 °C for 48 h. YPD broth was centrifuged at 5000× *g*, rinsed twice with sterile PBS, and wet cell pellets were stored in 100 mg aliquots at 4 °C. Yeast viability (CFU g^−1^) was checked at 0, 20, 50, and 65 days by plating tenfold dilutions on YPD agar supplemented with 0.05% chloramphenicol. 

### 2.19. Statistical Analysis

All experiments were performed in triplicate or duplicate (hydrophobicity). Results are expressed as the mean ± standard error. Statistical analyses were performed to detect significant differences (*p* ≤ 0.5) using one-way ANOVA after checking the assumptions of normality (Shapiro–Wilk test) and homogeneity of variance (Levene test). When significant differences were detected, the post-hoc HSD Tukey test (honestly-significant-difference) was performed. Non-normal data were transformed using the Johnson transformation for normalization or they were analyzed using the nonparametric Kruskal–Wallis test (Bonferroni method for *p*-value adjustment). In assays with cobia larvae, the Dunnett’s test was used to compare the effect of yeast with the control group. All tests were processed using R statistical software (version 4.2.1). Although all data were subjected to statistical tests for comparison between yeasts, the yeast selection process (in steps 1 and 2) was arbitrary. Selected yeasts had to have a biomass of greater than 1 g L^−1^, positive activity in more than 8 enzymes, negative or gamma hemolytic activity, an inhibition halo of greater than 1 mm in the antagonistic test, biofilm production of greater than 0.8 (OD), hydrophobicity, and autoaggregation of greater than 20%.

## 3. Results

### 3.1. Criteria to Select Probiotic Yeasts

In this study, we selected yeast strains based on their biotechnological, in vitro, and in vivo properties. Three yeast strains were identified as potential probiotics. This selection was performed in 3 steps (Figure S1). The selection process started with the isolation of 39 yeast colonies from intestinal mucosal cultures of adult cobia (n = 37). These 39 isolates were then identified by the sequencing of their ITS and 28S regions. They were then typed by RAPD-PCR to identify yeast strains (RAPD-PCR patterns with more than 85% similarity), and their dry biomasses were determined. Sixteen yeast strains were selected in this first step and subjected to the second selection step, where enzymatic, hemolytic, and antagonistic activities; biofilm production; hydrophobicity; and autoaggregation were evaluated. Yeast strains (n = 7) showing the best results moved to the third selection step, where we evaluated their polyamine production, their effect on larval survival, and their protective effect on hyposaline-stressed larvae.

### 3.2. Isolation and Identification of Yeasts Isolates from Cobia

Cobia fish were sampled from 2 Ecuadorian aquaculture centers (CENAIM and Emagrocom S.A.). Since our aim was to isolate diverse yeasts, we sampled fish fed different diets, considering that diet influences the diversity of microbiota (bacteria and fungi) [64]. From 37 cobia (with weights ranging from 327.6 to 5200.0 g), yeasts were detected in 100% of the fish, with a mean abundance of 6.76 ± 0.77 log_10_ CFU g^−1^ in the intestinal mucosa. Thirty-nine yeast isolates with different colony phenotypes in the YPD medium were selected. These first 39 yeast isolates corresponded to different colonies that we isolated in Petri dishes. These colonies were then purified and sequenced to identify the yeast species by sequencing their ITS and 28S regions. These 39 isolates were then subjected to RAPD-PCR typing. Yeasts of the same species that had identical RAPD patterns with >85% similarity were considered to represent a strain. It was possible to isolate more than one colony of the same strain from the same fish, but only one strain was selected for further analysis.

All yeast isolates identified within the genus *Debaryomyces* (Nucleotide BLAST NCBI) were assigned as *Debaryomyces* sp., as they showed high identity percentages with many *Debaryomyces* species. Therefore, of the 22 isolates assigned to *Debaryomyces* sp. by sequencing, 19 were identified as *Debaryomyces hansenii* by species-specific PCR (Table 1). Of all isolates, 97.4% were identified as Ascomycota (*Candida haemuloni, Candida parapsilosis, Debaryomyces hansenii, Debaryomyces* sp.) and 2.6% as Basidiomycota (*Naganishia* sp.). These 5 yeast species were all identified in Emagrocom S.A., whereas in CENAIM, only *C. haemuloni*, *D. hansenii,* and *Debaryomyces* sp. were identified. All yeast species were identified in cobia fed with frozen fish pieces, whereas only *D. hansenii* and *C. haemuloni* were detected in fish fed with formulated feed (CENAIM only) (Table S1).

### 3.3. First Selection of Yeasts by RAPD-PCR Profile and Biomass Production

**Figure 1:**
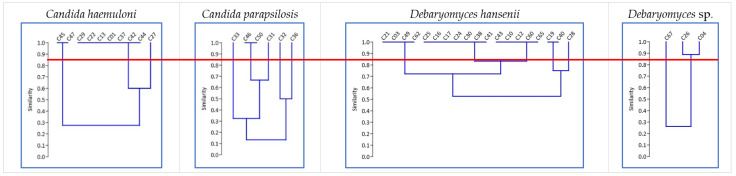
Dendrograms obtained from the RAPD-PCR of each yeast species by the UPGMA method (Jaccard distance). The red line shows a similarity level of 85%.

The RAPD-PCR profiles of the 39 isolates were analyzed to identify yeast strains. We detected 15 RAPD-PCR patterns with more than 85% similarity: 3 for *C. haemuloni*, 5 for *C. parapsilosis*, 5 for *D. hansenii*, and 2 for *Debaryomyces* sp. (Figure 1). The biomass production of these 39 isolates ranged from 0.07 ± 0.01 to 2.14 ± 0.17 g L^−1^ (Table 2). Yeasts belonging to the genus *Candida* produced higher biomasses (except for C47, C45, and C33), while *Naganishia* sp. and *Debaryomyces* sp. showed values of less than 1.05 g L^−1^. According to these results, we selected 15 yeast strains that showed unique RAPD profiles and the highest biomass production levels. We also selected the only yeast strain identified as *Naganishia* sp. In total, 16 yeast strains were selected in this step (Table 2).

### 3.4. Second Selection of Yeast Strains Based on their Enzymatic, Hemolytic, and Antipathogen Activity and their Potential to Adhere to Intestinal Mucosa

We determined hydrolytic enzymes in 16 yeast strains using the API ZYM system, which can detect a total of 19 enzymes. Eleven enzymes were detected (Table 3). Esterase (C4), esterase lipase (C8), leucine, and valine arylamidase were present in all yeast strains. The yeast strains producing the most enzymes were *C. parapsilosis* C31, C32, C46, and *D. hansenii* C10, which produced alkaline phosphatase and α glucosidase. The *C. haemuloni* C27, *D. hansenii* C10, and C28 strains produced lipase.

All 16 yeast strains showed γ hemolysis, meaning they were not hemolytic, and none of the yeast strains inhibited the growth of 5 pathogenic strains of *Vibrio*.

**Figure 2:**
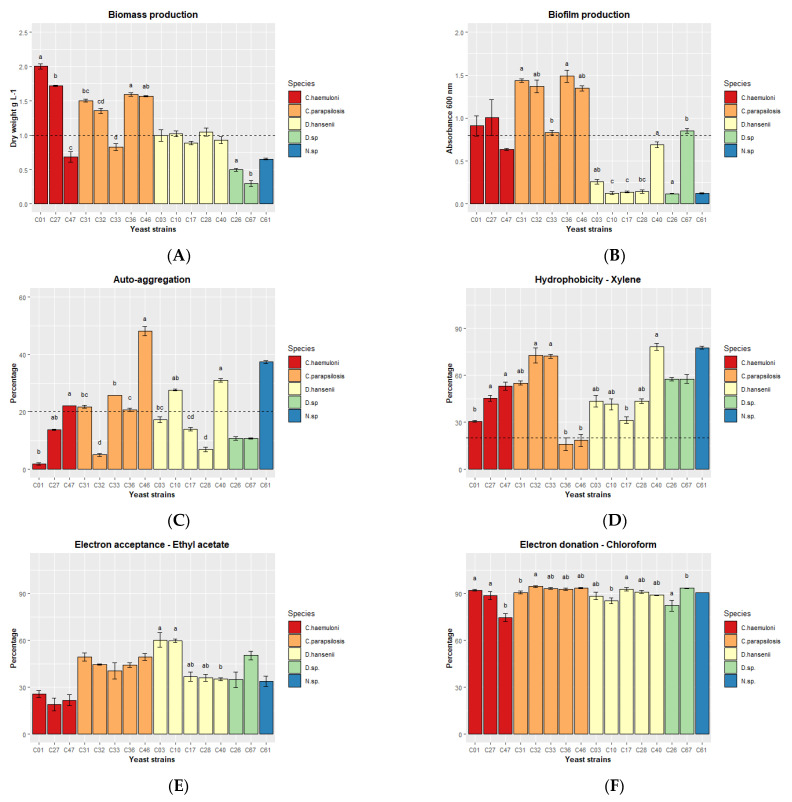
Different in vitro properties of the 16 yeast strains. (**A**) Biomass production (dry weight), (**B**) biofilm production, (**C**) autoaggregation at 1 h, (**D**) hydrophobicity, (**E**) electron acceptance, and (**F**) electron donation. The results are presented as the mean ± standard error. Different letters indicate significant differences (*p* < 0.05) between yeast strains of the same species. The dash line indicates the arbitrary criteria for the selection of yeasts.

Nine yeast strains—*C. haemuloni* (C01 and C27), *C. parapsilosis* (C31, C32, C36 and C46), and *D. hansenii* (C03, C10 and C28)—produced the highest biomasses, presenting dry weight values >1 g L^−1^ compared to the other strains (Figure 2A).

We then analyzed several in vitro tests related to the ability of a microorganism to interact with the intestinal mucosa: biofilm production, autoaggregation, hydrophobicity, and a test to identify electron donors vs. electron acceptors. All *C. parapsilosis, C. haemuloni* C01 and C27, and *Debaryomyces* sp. C67 produced biofilm after 48 h of cultivation (Figure 2B). Autoaggregation (> 20%) was detected after 1 h in 8 yeast strains: *C. haemuloni* C47; C. *parapsilosis* C31, C33, C36, and C46; *D. hansenii* C10 and C40; and *Naganishia* sp. C61 (Figure 2C). At 24 h, all yeast strains showed more than 78% autoaggregation (Table S2). Finally, all yeast strains, except *C. parapsilosis* C36 and C46, showed a medium hydrophobicity level (xylene, 30.68 ± 0.54 to 78.20 ± 2.21%) (Figure 2D). All yeast strains were medium and slow electron acceptors (ethyl acetate, 18.98 ± 4.08 to 60.26 ± 4.63%) (Figure 2E), and strong electron donors (chloroform, 74.53 ± 2.68 to 94.39 ± 0.66%) (Figure 2F) (Table S2).

According to the previous results, we selected the 7 yeast strains with the most characteristics of each species: two *C. haemuloni* (C01 and C27), three *C. parapsilosis* (C31, C32 and C46), and two *D. hansenii* (C10 and C28) (Table 4. Yeast strains C67 and C61 belong to different species; however, they were not considered because their biomass production was <0.7 g L^−1^. In addition, *C. parapsilosis* C36, although it had the same number of characteristics as C32 and C46, was excluded because it had the lowest enzymatic activity.

### 3.5. Third Selection Based on the Polyamine Production, Safety, and Protection of Larvae against a Saline Stress

**Figure 3:**
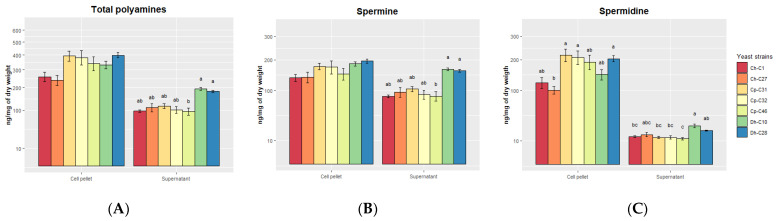
Polyamine production of yeast strains from the supernatants and cell pellets. (**A**) Total polyamines, (**B**) Spermine, and (**C**) Spermidine. The results are presented as the mean ± standard error. Ch, *C. haemuloni*; Cp, *C. parapsilosis*; Dh, *D. hansenii*; Dsp, *Debaryomyces* sp.; Nsp, *Naganishia* sp. Different letters indicate significant differences (*p* < 0.05) between yeast strains.

The polyamine concentration of the supernatants (extracellular) and yeast pellet cells (intracellular) of the 7 yeast strains was quantified (Figure 3). The cell pellets contained almost twice the concentration of polyamines (333.05 ± 75.94 ng mg^−1^ of dry weight) as the supernatants (180.07 ± 145.80 ng mg^−1^ of dry weight) (Figure 3A) (*p* < 0.001). In the cell pellets, there were no significant differences between yeast strains, while in the supernatants, *D. hansenii* C10 was shown to have a higher concentration than *C*. *haemuloni* C01 and *C. parapsilosis* C32 and C46. Spermine concentration in cell pellets did not differ between yeast strains, while in the supernatants, *D. hansenii* C10 and C28 produced higher concentrations compared with *C*. *haemuloni* C1 and *C. parapsilosis* C46 (Figure 3B). For spermidine, *C. parapsilosis* C31, C32 and *D. hansenii* C28 showed higher concentrations than *C*. *haemuloni* C27 in the pellet cells. In the supernatants, *D. hansenii* C10 showed a higher concentration than *C. parapsilosis* C46 (Figure 3C) (Table S3).

**Figure 4:**
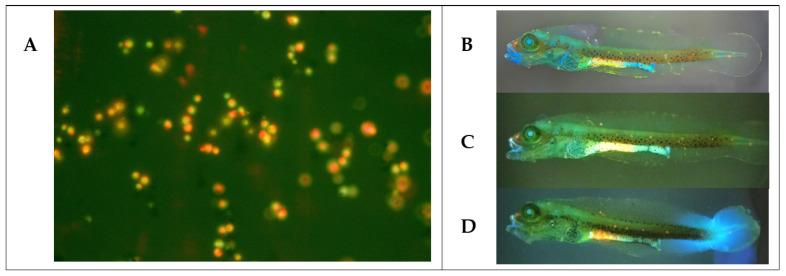
Yeast strains were observed in the intestines of cobia larvae after being inoculated by immersion with labeled yeast strains. (**A**) *D. hansenii* C10 stained with acridine orange (40×). (**B**) Cobia larvae (2 dph) after 1 h of being inoculated by immersion with stained *D. hansenii* C10, (**C**) *C. haemuloni* C27, and (**D**) *D. hansenii* C28 (4×).

**Figure 5:**
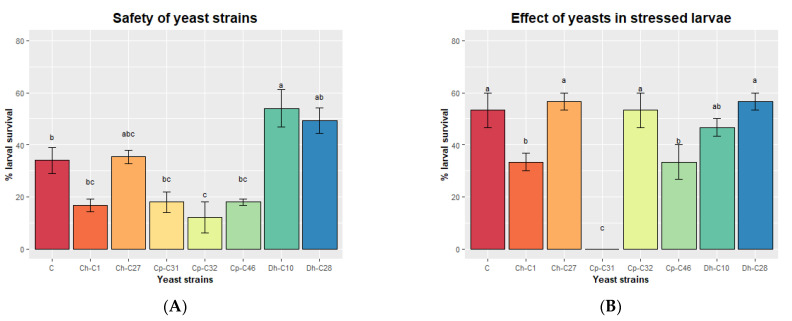
Effect of yeast strains on the survival of normal (**A**) and stressed larvae (**B**). (**A**) Larvae (2 dph) were exposed by immersion to yeast strains (10^7^ CFU mL^−1^), and survival was monitored for 24 h. (**B**) Larvae (4 dph) were fed with rotifers (*Brachionus rotundiformis*) enriched with each yeast strain. At 6 dph, larvae were transferred to hyposaline water (5 ppt), and survival was monitored for 8 h. Data are presented as the mean ± standard error. Ch, *C. haemuloni*; Cp, *C. parapsilosis*; Dh, *D. hansenii*; Dsp, *Debaryomyces* sp.; Nsp, *Naganishia* sp. Different letters indicate significant differences (*p* < 0.05) with the control group of larvae being nonexposed to yeasts.

Finally, we tested the safety of the yeast strains on cobia larvae. At 2 dph, larvae were inoculated by immersion with each yeast strain (10^7^ CFU mL^−1^) for 24 h, and larval survival was checked. All yeast strains entered the digestive tracts of the larvae, as fluorescent yeasts stained with acridine orange were detected in their intestines at 1 h after immersion inoculation (Figure 4). *C. parapsilosis* C32 was associated with reduced larval survival compared to the control group that was not inoculated with yeasts (Figure 5A). *C. haemuloni* C01 and C27, *C. parapsilosis* C31 and C46, and *D. hansenii* C28 did not affect survival. Interestingly, larvae inoculated with *D. hansenii* C10 showed a higher survival rate (54.0 ± 7.21%), 20% more than the control group, which was not inoculated with yeasts. We then tested the effect of yeast strains on stressed larvae. At 4 dph, larvae were fed rotifers enriched with each yeast strain. At 6 dph, larvae were transferred to hyposaline water (5 ppt), and survival was recorded after 8 h. Larvae fed with C27, C32, C10, or C28 showed the same survival rates as control larvae fed with nonenriched rotifers. However, larvae fed with rotifers enriched with *C. haemuloni* C01 and *C. parapsilosis* C31 and C46 showed lower survival rates than the control group (Figure 5B) (Table S4).

According to these results, 3 potential probiotic yeast strains (*C. haemuloni* C27 and *D. hansenii* C10 and C28) were selected and further characterized.

### 3.6. Characterization of Selected Potential Probiotic Yeast Strains

**Figure 6:**
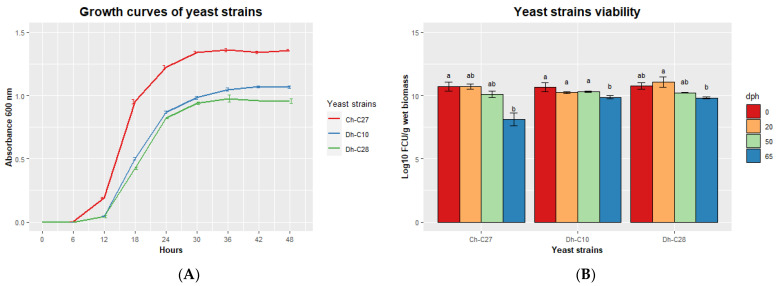
(**A**) Growth curves of selected yeast strains over 48 h at 28 °C and (**B**) viability of wet pellets of yeast strains stored at 4 °C at 0, 20, 50, and 65 dph (days post-harvest). Different letters indicate significant differences (*p* < 0.05).

We analyzed the growth curves of the 3 selected yeast strains (Figure 6A). *C*. *haemuloni* C27 reached maximum absorbance (1.34 ± 0.02) at 30 h, while *D*. *hansenii* C10 (1.05 ± 0.02) and C28 (0.98 ± 0.05), reached maximum absorbance at 36 h. The 3 yeast strains reached a concentration of 1 × 10^10^ CFU g^−1^ of wet biomass at 48 h. This concentration remained constant for 50 days for all yeast strains. However, after 65 days, the yeast viability was reduced for all yeast strains, especially for *C*. *haemuloni* C27, for which the viability decreased to 8.11 ± 0.71 log_10_ CFU g^−1^ wet biomass (Figure 6B).

## 4. Discussion

The continuous growth of aquaculture, the intensification of production demand, and the improvement of the efficiency of farming systems are forcing the industry to increase production in a sustainable way. Several alternatives have been studied in the last decades, including the use of probiotics. Potential probiotic bacteria have been isolated from cobia in previous studies [14]. However, the numerous properties that yeasts possess [65] make them very attractive for use in this fish species at different stages of development.

We identified and selected potential probiotic yeast strains from the intestinal mucosa of cobia (*Rachycentron canadum*) of different sizes and feeding regimes sampled from 2 facilities. No previous studies have been done on the autochthonous mycobiota of cobia. Yeasts were present in all fish, as reported in other marine species [26,36,43]. The yeast concentration (6.76 ± 0.77 log_10_ CFU g^−1^ intestinal mucosa) was similar to that described for other fishes [27] but higher than that of some carnivorous marine fishes from Chile, which have been reported to have concentrations ranging from 2.32 to 4.22 log_10_ CFU g^−1^ [26]. Considering that the optimum growth temperature for yeasts is between 25 and 30 °C and that the Chilean fish species live at temperatures of around 15 °C, and cobia live at 28 °C, this could be a factor that explains the differences observed, in addition to the differences in the host species and the type of sample (intestinal mucosa *versus* intestinal content) employed [66].

We identified *Candida* spp. and *Debaryomyces* spp. from the Ascomycota phylum and *Naganishia* sp. (formerly *Cryptococcus*) from the Basidiomycota phylum. These genera have been reported to be part of the microbiota of healthy fish [26,27,31,36,43,67,68]. In particular, at the species level, *Debaryomyces hansenii* has been widely identified in the gut mycobiota of healthy fish [26,27,31,65]. *C. haemuloni* was first described from the intestine of a marine fish (*Haemulon scirus*) in 1962 [69]. Although yeasts of the genus *Candida* have been reported to be pathogenic organisms, they are also present in the intestinal microbiota of healthy fish [27]. In this study, fish fed frozen fish pieces had greater diversity in terms of cultured yeasts than fish fed formulated feed. The effect of diet on the fish gut microbiota has been observed in numerous studies [64]. However, no studies on cultured fungal communities have been reported.

In this study, all yeast isolates were screened for various properties that have been recommended for the in vitro selection of potential probiotics [70] and used to characterize yeast strains isolated from marine fish [26,28,34,36,40,55]. Our selection was based on three steps. The first step consisted of yeast strain identification (RAPD-PCR) and biomass production. We evaluated biomass production in the first selection step, because this biotechnological property can be a bottleneck for the production of probiotics in large-scale aquaculture. The yeast isolates reached a dry biomass production level of between 0.3 and 2.0 g L^−1^ when they were grown in a batch with a commercial culture medium (YPD) in 15 mL tubes. It is worth noting that production could be improved by increasing the aeration conditions or by using alternative carbon sources, such as beet or sugar cane molasses [71], hydrolyzed lignocellulosic biomass, or animal byproducts [72]. At the same time, other technologies can increase the biomass yield of a fed-batch culture [71], such as continuous feeding in a bioreactor or protein and strain engineering using molecular tools [73]. Another factor that can be optimized to increase the biomass is the temperature. However, we only evaluated the yeast biomass production at the culture temperature of cobia (28 °C) to select strains with advantageous growth characteristics at this host culture temperature [70]. This led us to hypothesize that these yeast strains could be used in other tropical farmed fish, although further studies are needed.

In the second selection step, we analyzed the ability of the yeast strains to produce hydrolytic enzymes, to have antipathogen activity, and to potentially adhere to the intestinal mucosa. This is similar to what has been done in other studies [26,28,34,36,40,55]. We measured the activity of 19 hydrolytic enzymes (esterases, proteases, lipases, and glycosidases) that can promote the digestive process [74]. The enzymatic activity profile of yeasts is highly variable [75]. However, the enzymes present in all yeast strains in this study were esterase (C4), esterase lipase (C8), leucine arylamidase, and valine arylamidase. These enzymes have also been reported in all yeast strains isolated from the marine fish *Genypterus chilensis* and *Seriolella violacea* [36] and in more than 85% of yeast strains isolated from *Salmo salar, Oncorhynchus kisutch*, *O. mykiss*, *Seriola lalandi,* and *Cilus gilberti* [26]. The same profile has been observed in yeasts of medical importance [75] and yeasts isolated from environmental samples [76]. In general, the enzymatic profiles of the yeast strains isolated from cobia were similar to those reported for yeasts from the marine fish mentioned above. This can be explained by the fact that the diets of these marine fish are similar with high protein and lipid contents and a low carbohydrate content [26,77]. Aminopeptidases such as leucine arylamidase, valine arylamidase, and cystine arylamidase were present in most of the yeast strains. Lipase and/or esterase lipase were present in all yeast strains, while a few glycosidases were detected. Alpha-glucosidase was present in all yeast strains of *C. parapsilosis* and *D. hansenii* C10 and rarely found in yeasts from marine fish [26,36]. Beta-glucosidase is an enzyme with biotechnological potential, because it can degrade cellulose [78]. In our study, this enzyme was present in only two yeast strains, which could be due to the carnivorous habits of the cobia. Phosphatases (acid or alkaline phosphatase) were detected in all yeast strains. Acid phosphatase and naphthol-AS-BI-phosphohydrolase were the most prevalent, similar to results reported for other yeasts from marine carnivorous fish [26,36]. Interestingly, alkaline phosphatase can dephosphorylate and detoxify the endotoxin component of bacterial lipopolysaccharides (LPS), which induce inflammatory responses in the host [79]. Therefore, these enzymes may play an important role in the metabolism of the host, as they are related to phosphorus and carbon cycles, can supply phosphorus to the host and other microorganisms [77], and reduce the bacterial pathogenicity in the gut [79].

None of the yeast strains showed antibacterial activity against the 5 pathogenic strains (*Vibrio harveyi* E22, *V. campbellii* LM2013, *V. parahemolyticus* BA94C2, *V. vulnificus* S2, and *V. anguillarum* PF4). Few studies have reported on the antimicrobial activity of yeasts against aquaculture pathogens, such as *Sporidiobolus ruineniae* A45.2, which has been shown to have antagonistic activity against *Bacillus cereus* TISTR 747, *Staphylococcus aureus* TISTR 746, and *Streptococcus agalactiae* DMST 11366 [80]. Moreover, a commercial product, Yeast Glycoproteins (which mainly contained mannan oligosaccharide (≥12%), β-dextran (≥12%), H_2_O (≤6%), and crude protein (≤35%), etc.), showed in vitro antibacterial activity against *Aeromonas caviae* (a pathogenic bacterium of *Carassius auratus gibelio*) [81].

The ability of fungal species to attach to and grow on different substrates or hosts is surprisingly broad [82]. When attached to the intestinal mucosa, they protect the host from pathogen colonization by competing for host cell binding sites [83]. In this study, 3 in vitro assays (hydrophobicity, autoaggregation, and biofilm production) associated with gut adherence and colonization [84,85] were performed. Hydrophobic cells are more resistant to phagocytic killing and they adhere more easily to host tissue and to the gastrointestinal mucosa, which also has hydrophobic properties [86,87]. In our study, the hydrophobicity of the yeast strains ranged from 16 to 78%, which is similar to the values observed for probiotic yeasts isolated from table olives (12 to 90%) [88]. Additionally, in this study, *C. parapsilosis* (CCMA 1777, 1756) [88] and C. *parapsilosis* (C36 and C46) showed the lowest hydrophobicity values. We also examined the adherence to other organic solvents, such as chloroform and ethyl acetate, to determine the ability of the yeast strains to be electron donors and electron acceptors, respectively [59]. Since all yeast strains showed greater affinity for chloroform compared to ethyl acetate, they were all classified as electron donors and weak electron acceptors. This result is similar to that found for two fouling bacteria isolated from the dairy industry, *Streptococcus thermophilus* B and *Leuconostoc mesenteroides* NCDO 523 [59], but is different from the results for potential probiotic bacteria (*Bacillus* sp. RCS1, *Pantoea agglomerans* RCS2, and *Bacillus cereus* RCS3) isolated from cobia [14] and *Bacillus* strains (GPSAK2, GPSAK9, and GPSAK4) isolated from the hybrid grouper (*Epinephelus fuscoguttatus*♀ *× Epinephelus lanceolatus*♂) [52], which showed a high capacity to both accept and donate electrons. To date, no studies have reported the behavior of yeasts with these organic solutions. The presence of carboxylic groups on the microbial surface can explain this Lewis acid–base interaction between a microorganism and a surface [59].

Biofilms are formed by the aggregation of microorganisms into multicellular structures that adhere to surfaces [89]. In this study, *Debaryomyces* and *Naganishia* strains were shown to have a low ability to form a biofilm, in contrast to those of the genus *Candida*. *Candida* species have been studied for their medical relevance and show a high ability to form biofilm [90]. Beneficial yeast biofilms have been described in the food industry and are mostly associated with *Saccharomyces cerevisiae* [82].

In this study, at least one strain of each genus autoaggregated after 1 h, and all yeast strains autoaggregated after 24 h, similar to the results reported for probiotic yeasts from kefir [91], pathogenic *Candida* spp., and probiotic yeast *Saccharomyces boulardii* [92]. Adhesion in yeasts is mediated by specialized cell-surface proteins called adhesins or flocculins that bind to specific amino acids or sugar residues on the surfaces of other cells or abiotic surfaces [90]. Autoaggregation is a cell–cell adhesion process that can facilitate yeast adhesion to the mucosa, thereby promoting the colonization of the intestine. Conversely, a recent study showed that an autoaggregating strain was cleared more rapidly from the intestine by peristaltic movements compared to nonaggregating strains [93]. This highlights the importance of performing in vivo studies to predict the colonization ability of a microorganism and shows some limitations of our study.

Finally, yeast strains were selected based on their polyamine production and positive effects on cobia larvae. Polyamines are ubiquitous molecules that are involved in cellular metabolism and protein, RNA, and DNA synthesis [94] and are considered to be essential growth factors [95]. These molecules are found in every living cell, and the diet can also provide sufficient amounts to support cell renewal and growth [94]. Many studies have shown that spermine improves enterocyte maturation both in mammals [94,96] and in fish, such as sea bass (*Dicentrarchus labrax*) [97]. On this basis, polyamine-producing yeasts have been characterized [37] and used in the early stages of fish to improve digestive [38,44,98,99] and immunological maturation [42,100]. In this study, spermine and spermidine were detected in 7 yeast strains, and no yeast strain produced putrescine. The concentrations of total polyamines (spermine + spermidine) in the cell pellets were higher than in the supernatant, and no differences were observed between yeast strains. On the contrary, higher concentrations of polyamines were detected in the supernatants of *D. hansenii* HF1 (CBS8339) and *S. cerevisiae* X2180 cultured in YPD broth until the early stationary phase [38]. This difference could be attributed to polyamines present in the YPD culture medium, since this medium contains yeast extract. Therefore, we cultured our yeast strains in a minimal medium without polyamines. In our study, the amounts of spermidine and spermine in the cell pellets were similar. However, in the supernatant, spermine was almost 7 times more concentrated than spermidine, suggesting that yeasts secrete more spermine. In contrast, *D. hansenii* HF1 (CBS8339) secreted similar amounts of spermidine and spermine, and in the cell pellet, the spermidine concentration was nearly three times higher than that of spermine [44]. Considering that the quantification methods and yeast growth conditions differed between studies, we did not compare the extent of polyamine production. We note that all yeast strains were able to secrete a greater amount of spermine compared to spermidine, which has been shown to have beneficial effects at the digestive level [97]. Spermidine also has other properties, such as antiaging, anti-inflammatory, and antioxidant effects. Spermidine inhibits the production of proinflammatory mediators and reduces the accumulation of reactive oxygen species (ROS) in RAW 264.7 macrophages and zebrafish (*D. rerio*) larvae induced with LPS [101].

Safety testing is one of the essential requirements for probiotic candidates [70]. In this study, hemolytic activity on cobia blood agar showed that all yeast strains were unable to lyse cobia cell blood. In addition, the 3 selected yeast strains did not affect larval survival when they were inoculated by immersion. Additionally, larvae inoculated with *D. hansenii* C10 showed a higher survival rate compared with that of noninoculated control larvae. The 3 selected yeast strains also did not affect the survival of larvae exposed to hyposaline stress.

Finally, we selected 3 yeast strains as potential probiotics: *D. hansenii* C10 and C28 and *C. haemuloni* C27. Further in vivo studies in cobia are needed to confirm their probiotic effects. Other probiotic yeast strains from the same yeast species have also been identified. To date, the most studied strain is *D. hansenii* CBS8339, isolated from a rainbow trout (*Salmo gairdneri*) [31]. In sea bass (*D. labrax*) larvae and juveniles, this strain was able to adhere to the intestinal epithelium, increase digestive enzyme activity, reduce he malformations, modulate the antioxidant activity, and increase fish survival [33,38,39,99]. In gilthead seabream (*Sparus aurata*) juveniles, *D. hansenii* CBS8339 stimulated the innate immune response [100]. In longfin yellowtail (*Seriola rivoliana*) larvae, *D. hansenii* CBS8339 stimulated digestive tract maturation, survival, growth, and bone mineralization and reduced skeletal deformities [98]. Likewise, feeding *D. hansenii* CBS8339 to juvenile leopard grouper (*Mycteroperca rosacea*) improved their immune response and resistance to the protozoan *Amyloodinium ocellatum* [100] and increased their growth performance, antioxidant activity, and immunological response to the bacterial pathogen *Aeromonas hydrophila* [42]. Similarly, the strain *D. hansenii* 97, isolated from a rainbow trout (*Oncorhynchus mykiss*), has shown a probiotic effect in zebrafish (*Danio rerio*). This strain was shown to protect zebrafish larvae against *V. anguillarum* PF4 infection by preventing the expression of inflammatory cytokines IL1β and TNFα [40]. On the other hand, the *C*. *haemuloni* S27 strain, isolated from seawater and administered to the giant tiger shrimp (*Penaeus monodon*), reduced mortality caused by white spot syndrome virus (WSSV) and increased the expression of antimicrobial peptides (AMPs), which confer better protection against WSSV [102].

An important criterion for a probiotic is its ability to maintain its viability during storage [70]. For this purpose, we measured the viability of the wet cell pellets stored at 4 °C for up to 50 days. We chose these conditions, because these storage conditions can be easily adopted at the farm level, as they do not require sophisticated equipment, such as a −80 °C freezer, or complex methods, such as lyophilization. However, it is worth noting that we can increase the viability time of a microorganism by using freezing, vacuum drying, or lyophilization with or without the use of protective agents [103,104,105,106]. Among these methods, lyophilization is the most convenient and successful way to preserve microorganisms; however, not all strains can survive the process [103]. This is the case for 95 strains of *Saccharomyces cerevisiae*, whose viability decreases to 10% immediately after desiccation, although the viability has been shown to be maintained during a 10-year storage period [107]. Further studies are needed to develop a standardized method to increase the storage times of the 3 selected yeast strains.

## 5. Conclusions

We selected three yeast strains with probiotic potential. *C. haemuloni* C27, *D. hansenii* C10, and C28 possess desirable characteristics based on their biomass production, ability to adhere to the intestine, enzymatic activity, safety, protective effect against a hyposaline stress, and polyamine production. However, this study is a preliminary approach, and additional in vivo assays should be performed to verify the colonization capacity and probiotic effect of each strain in the different developmental stages of cobia or other fish species.

## Figures and Tables

**Table 1:** Identification of yeast isolates cultured from the intestinal mucosa of cobia (*R. canadum*).

Fish Characteristics				Characteristics of Yeast Isolates							
Origin	Weight (g)	Feed	Fish Code	N°	Yeast Isolate Code	Sequence Length (bp)	Closest Relatives	Identity (%)	Phylum	Species Assigned by Specific Primers	NCBI Accession Codes
E	1447.8	FFP	FC2	1	C29	860	*Candida haemuloni* JX459773.1	99.19	A		*Candida haemuloni* OQ184038
				2	C31	1059	*Candida parapsilosis* MH545914.1 and MK394125.1	99.62	A		*Candida parapsilosis* OQ184039
E	1410.2	FFP	FC4	3	C47	874	*Candida haemuloni* JX459773.1	99.31	A		*Candida haemuloni* OQ184040
E	1271.2	FFP	FC6	4	C32	1044	*Candida parapsilosis* MH545914.1 and MK394125.1	99.52	A		*Candida parapsilosis* OQ184041
				5	C36	513	*Candida parapsilosis* KX652404.1	99.22	A		*Candida parapsilosis* OQ184042
				6	C46	1058	*Candida parapsilosis* MH545914.1	99.06	A		*Candida parapsilosis* OQ184043
				7	C50	1045	*Candida parapsilosis* MH545914.1 and MK394125.1	97.63	A		*Candida parapsilosis* OQ184044
E	1087.6	FFP	FC3	8	C62	1073	*Debaryomyces fabryi* MK394103.1, and *Debaryomyces hansenii* KX981201.1	99.24	A	*D. hansenii*	*Debaryomyces* sp. OQ184045
E	871.8	FFP	FC5	9	C27	861	*Candida haemuloni* JX459773.1	99.42	A		*Candida haemuloni* OQ184046
				10	C24	1175	*Debaryomyces hansenii* FR686594.1	98.98	A	*D. hansenii*	*Debaryomyces* sp. OQ184047
				11	C28	1130	*Debaryomyces hansenii* FR686594.1 and GQ458025.1	98.41	A	*D. hansenii*	*Debaryomyces* sp. OQ184048
E	801.8	FFP	FC1	12	C37	878	*Candida haemuloni* JX459773.1	99.43	A		*Candida haemuloni* OQ184049
				13	C33	993	*Candida parapsilosis* MZ375521.1 and MZ375508.1	97.79	A		*Candida parapsilosis* OQ184050
				14	C25	1154	*Debaryomyces hansenii* FR686594.1 and GQ458025.1	98.70	A	*D. hansenii*	*Debaryomyces* sp. OQ184051
				15	C30	1149	*Debaryomyces hansenii* FR686594.1 and GQ458025.1	97.05	A	*D. hansenii*	*Debaryomyces* sp. OQ184052
				16	C38	1091	*Debaryomyces fabryi* MK394103.1, and *Debaryomyces hansenii* KC111444.1	99.91	A	*D. hansenii*	*Debaryomyces* sp. OQ184053
				17	C26	1175	*Debaryomyces hansenii* FR686594.1	98.56	A		*Debaryomyces* sp. OQ184054
				18	C67	1156	*Debaryomyces hansenii* FR686594.1 and GQ458025.1	98.45	A		*Debaryomyces* sp. OQ184055
				19	C61	978	*Naganishia adeliensis* KY218697.1, and *Naganishia* sp. LC529181.1	97.75	B		*Naganishia* sp. OQ184056
C	5200.0	FFP	FC32	20	C13	842	*Candida haemuloni* JX459773.1	99.17	A		*Candida haemuloni* OQ184057
				21	C16	1157	*Debaryomyces hansenii* FR686594.1 and GQ458025.1	97.84	A	*D. hansenii*	*Debaryomyces* sp. OQ184058
				22	C17	858	*Debaryomyces fabryi* MK394103.1, and *Debaryomyces hansenii* KC111444.1	98.26	A	*D. hansenii*	*Debaryomyces* sp. OQ184059
C	4780.0	FFP	FC34	23	C01	674	*Candida haemuloni* OW987803.1	94.51	A		*Candida haemuloni* OQ184060
C	511.2	FFP	FC20	24	C03	1154	*Debaryomyces hansenii* FR686594.1 and GQ458025.1	99.39	A	*D. hansenii*	*Debaryomyces* sp. OQ184061
				25	C40	880	*Debaryomyces fabryi* MK394103.1, and *Debaryomyces hansenii* KX981201.1	98.86	A	*D. hansenii*	*Debaryomyces* sp. OQ184062
				26	C41	1026	*Debaryomyces fabryi* MK394103.1, *Debaryomyces hansenii* KX981201.1, and *Debaryomyces* sp. MW959732.1	99.81	A	*D. hansenii*	*Debaryomyces* sp. OQ184063
				27	C04	1094	*Debaryomyces fabryi* MK394103.1, and *Debaryomyces hansenii* KX981201.1	99.91	A		*Debaryomyces* sp. OQ184064
C	498.6	FFP	FC23	28	C44	847	*Candida haemuloni* JX459773.1	99.41	A		*Candida haemuloni* OQ184065
				29	C45	863	*Candida haemuloni* JX459773.1	99.42	A		*Candida haemuloni* OQ184066
				30	C10	1159	*Debaryomyces hansenii* FR686594.1 and GQ458025.1	99.13	A	*D. hansenii*	*Debaryomyces* sp. OQ184067
C	489.2	FFP	FC24	31	C21	1022	*Debaryomyces fabryi* MK394103.1, and *Debaryomyces hansenii* KX981201.1	97.36	A	*D. hansenii*	*Debaryomyces* sp. OQ184068
C	410.4	FFP	FC25	32	C43	1151	*Debaryomyces hansenii* FR686594.1 and GQ458025.1	97.93	A	*D. hansenii*	*Debaryomyces* sp. OQ184069
				33	C65	1141	*Debaryomyces hansenii* FR686594.1 and GQ458025.1	98.87	A	*D. hansenii*	*Debaryomyces* sp. OQ184070
C	370.6	FF	FC28	34	C19	1152	*Debaryomyces hansenii* FR686594.1 and GQ458025.1	98.35	A	*D. hansenii*	*Debaryomyces* sp. OQ184071
C	366.8	FF	FC27	35	C22	853	*Candida haemuloni* JX459773.1	98.59	A		*Candida haemuloni* OQ184072
				36	C42	365	*Candida haemuloni* MH667577.1 and MH636864.1	100.00	A		*Candida haemuloni* OQ184073
				37	C12	1160	*Debaryomyces hansenii* HE799666.1 and HE799660.1	97.76	A	*D. hansenii*	*Debaryomyces* sp. OQ184074
				38	C60	1190	*Debaryomyces hansenii* FR686594.1	96.83	A	*D. hansenii*	*Debaryomyces* sp. OQ184075
C	327.6	FF	FC30	39	C49	1154	*Debaryomyces hansenii* FR686594.1 and GQ458025.1; and *Debaryomyces* sp. MW959725.1	98.43	A	*D. hansenii*	*Debaryomyces* sp. OQ184076

Origin: C, CENAIM; E, Emagrocom S.A. Feed: FFP, frozen fish pieces; FF, formulated feed. Phylum: A, Ascomycota; B, Basidiomycota.

**Table 2:** RAPD-PCR profiles and biomass production of yeast isolates. Yeasts marked with ✓ were selected. The results are presented as the mean ± standard error.

N°	Yeast Isolate Code	Species	Dry Weight (g L^−1^)	RAPD-PCR Pattern	Selected Yeast Strains
1	C47	*Candida haemuloni*	0.69 ± 0.13	A	✓
2	C45	*Candida haemuloni*	0.57 ± 0.07	A	
3	C44	*Candida haemuloni*	2.14 ± 0.17	B	
4	C01	*Candida haemuloni*	2.00 ± 0.07	B	✓
5	C13	*Candida haemuloni*	1.94 ± 0.05	B	
6	C29	*Candida haemuloni*	1.89 ± 0.06	B	
7	C37	*Candida haemuloni*	1.86 ± 0.08	B	
8	C22	*Candida haemuloni*	1.84 ± 0.10	B	
9	C42	*Candida haemuloni*	1.65 ± 0.03	B	
10	C27	*Candida haemuloni*	1.72 ± 0.01	C	✓
11	C33	*Candida parapsilosis*	0.83 ± 0.09	D	✓
12	C46	*Candida parapsilosis*	1.57 ± 0.02	E	✓
13	C50	*Candida parapsilosis*	1.53 ± 0.04	E	
14	C31	*Candida parapsilosis*	1.50 ± 0.04	F	✓
15	C32	*Candida parapsilosis*	1.36 ± 0.07	G	✓
16	C36	*Candida parapsilosis*	1.59 ± 0.04	H	✓
17	C28	*Debaryomyces hansenii*	1.05 ± 0.06	I	✓
18	C40	*Debaryomyces hansenii*	0.93 ± 0.09	J	✓
19	C19	*Debaryomyces hansenii*	0.88 ± 0.06	J	
20	C03	*Debaryomyces hansenii*	1.00 ± 0.15	K	✓
21	C21	*Debaryomyces hansenii*	0.86 ± 0.08	K	
22	C49	*Debaryomyces hansenii*	0.78 ± 0.07	K	
23	C62	*Debaryomyces hansenii*	0.07 ± 0.01	K	
24	C38	*Debaryomyces hansenii*	0.98 ± 0.13	L	
25	C17	*Debaryomyces hansenii*	0.89 ± 0.04	L	✓
26	C24	*Debaryomyces hansenii*	0.88 ± 0.05	L	
27	C30	*Debaryomyces hansenii*	0.87 ± 0.08	L	
28	C25	*Debaryomyces hansenii*	0.86 ± 0.02	L	
29	C16	*Debaryomyces hansenii*	0.83 ± 0.08	L	
30	C41	*Debaryomyces hansenii*	0.70 ± 0.12	L	
31	C10	*Debaryomyces hansenii*	1.02 ± 0.08	M	✓
32	C65	*Debaryomyces hansenii*	0.95 ± 0.06	M	
33	C12	*Debaryomyces hansenii*	0.94 ± 0.09	M	
34	C43	*Debaryomyces hansenii*	0.93 ± 0.04	M	
35	C60	*Debaryomyces hansenii*	0.91 ± 0.07	M	
36	C26	*Debaryomyces* sp.	0.87 ± 0.06	N	✓
37	C04	*Debaryomyces* sp.	0.50 ± 0.04	N	
38	C67	*Debaryomyces* sp.	0.30 ± 0.07	O	✓
39	C61	*Naganishia* sp.	0.66 ± 0.02	P	✓

**Table 3:** Enzymatic activity of the 16 yeast strains determined using the API-ZYM colorimetric test.

N°	Enzymes	*Candida* *haemuloni*			*Candida parapsilosis*					*Debaryomyces hansenii*					*Debaryomyces* sp.		*Naganishia* sp.
		C01	C27	C47	C31	C32	C33	C36	C46	C03	C10	C17	C28	C40	C26	C67	C61
1	Alkaline phosphatase	-	-	-	±	±	-	±	±	±	±	±	±	-	-	-	-
2	Esterase (C 4)	+	±	±	±	±	±	±	+	±	±	±	±	±	±	±	±
3	Esterase Lipase (C 8)	+	±	±	±	±	±	±	±	±	±	±	±	±	±	±	±
4	Lipase (C 14)	-	±	-	-	-	-	-	-	-	±	-	±	-	-	-	-
5	Leucine arylamidase	+	+	+	+	+	+	+	+	±	±	+	+	+	+	+	+
6	Valine arylamidase	+	+	±	+	+	±	+	+	±	±	±	±	±	±	±	±
7	Cystine arylamidase	±	±	-	±	±	-	-	±	-	-	-	-	-	-	-	-
8	Phosphatase acid	-	±	+	+	+	±	+	+	-	±	±	±	±	±	±	+
9	Naphthol-AS-BI-phosphohydrolase	-	±	±	±	+	±	+	+	-	±	±	±	±	±	±	±
10	α glucosidase	-	-	-	±	±	±	±	+	-	±	-	-	-	-	-	-
11	β glucosidase	-	-	+	-	-	-	-	-	-	-	-	-	-	-	-	+
	Total enzymes	5	8	7	9	9	7	8	9	5	9	7	8	6	6	6	7

Enzymatic activity: + high; ± middle; - no activity.

**Table 4:** Characteristics of selected yeast strains (checked) according to their biomass production, hemolysis, and enzymatic activity; antipathogen effect; and their potential to adhere to the intestinal epithelium.

Yeast strains			Safety	Action against Pathogens	Potential to Adhere to Intestinal Mucosa				
	Biomass Production	Enzymatic Activity	Hemolysis of Cobia Erythrocytes	Antagonism against *Vibrio* spp.	Biofilm	Autoaggregation	Hydrophobicity	Total	Selected Yeast Strains
Ch−C01	+	-	-	-	+	-	+	3	✓
Ch−C27	+	+	-	-	+	-	+	4	✓
Ch−C47	-	-	-	-	-	+	+	2	
Cp−C31	+	+	-	-	+	+	+	5	✓
Cp−C32	+	+	-	-	+	-	+	4	✓
Cp−C33	-	-	-	-	+	+	+	3	
Cp−C36	+	+	-	-	+	+	-	4	
Cp−C46	+	+	-	-	+	+	-	4	✓
Dh−C03	+	-	-	-	-	-	+	2	
Dh−C10	+	+	-	-	-	+	+	4	✓
Dh−C17	-	-	-	-	-	-	+	1	
Dh−C28	+	+	-	-	-	-	+	3	✓
Dh−C40	-	-	-	-	-	+	+	2	
Dsp−C26	-	-	-	-	-	-	+	1	
Dsp−C67	-	-	-	-	+	-	+	2	
Nsp−C61	-	-	-	-	-	+	+	2	

Ch, C. *haemuloni*; Cp, C. *parapsilosis*; Dh, D. *hansenii*; Dsp, *Debaryomyces* sp.; Nsp, *Naganishia* sp. Biomass production > 1 g L^−1^; production of at least 8 enzymes; inhibition halo >1 mm; autoaggregation at 1 h; hydrophobicity > 20%; and biofilm production (Absorbance >0.8) were considered positive characteristics.

## Data Availability

All sequences obtained from yeast isolates were submitted to the GenBank dataset under the accession numbers OQ184038–OQ184076.

## Supplementary Materials

The following supporting information can be downloaded at: https://www.mdpi.com/article/10.3390/jof9020274/s1, Table S1. Yeast isolates (n = 39) from cobia fish (n = 37; fish code). Table S2. Results of the second selection step of yeast strains. Table S3. Polyamine quantification in the cell pellets and supernatants of the 7 yeast strains. Table S4. Survival of cobia larvae in safety and protection experiments. Figure S1. Flow chart showing the selection process performed to identify yeast strains with probiotic potential.
