# Supplementary material for: Selection of Autochthonous Yeasts Isolated from the Intestinal Tracts of Cobia Fish (Rachycentron canadum) with Probiotic Potential
Source: J Fungi (Basel). 2023 Feb 18;9(2):274. doi: 10.3390/jof9020274 (PMC9966584; doi:10.3390/jof9020274)
Supplement: Supplementary file 1 [file jof-09-00274-s001.zip › Supplementary material/Figure S1_rev1.pdf]

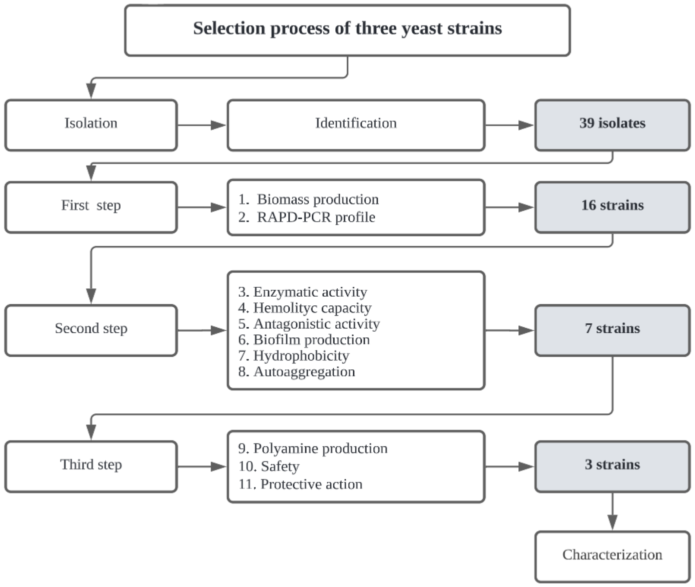

Figure 1. Flow chart showing the selection process performed to identify yeast strains with probiotic potential.
