# Supplementary material for: Selection of Autochthonous Yeasts Isolated from the Intestinal Tracts of Cobia Fish (Rachycentron canadum) with Probiotic Potential
Source: J Fungi (Basel). 2023 Feb 18;9(2):274. doi: 10.3390/jof9020274 (PMC9966584; doi:10.3390/jof9020274)
Supplement: Supplementary file 1 [file jof-09-00274-s001.zip › Supplementary material/Table S1_rev1.pdf]

Table S1. Yeast isolates (n=39) from cobia fish (n=37; fish code).

	Samples										Isolates						
**N°**	**Origin**	**Fish code**	**Weight (g)**	**Sex**	**Fish**	**Feed**	**T (°C)**	**Salinity (ppt)**	**Sample weight (g)**	**Sample code**	**Isolate code**	**Cell morphology**	**Type**	**Species**	**RAPD profile**	**Colonies in petri plate 48 hours**	**Cell morphology**
1	C	C34	4780.0	male	*Rachycentron canadum*	FFP	27	35	0.35	34	**C01**	oval	yeast	*Candida haemuloni*	B	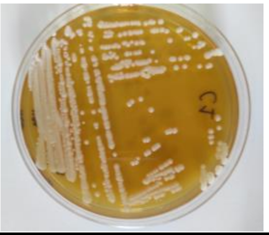	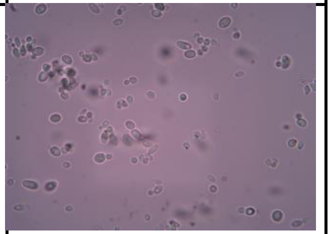
2	C	C20	511.2	IND	*Rachycentron canadum*	FFP	27	35	0.55	20	**C03**	round	yeast	*Debaryomyces* sp.	K	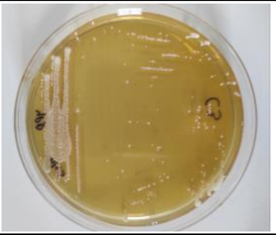	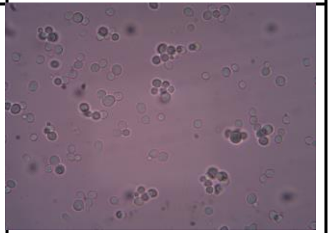
3	C	C20	511.2	IND	*Rachycentron canadum*	FFP	27	35	0.55	20	**C04**	round	yeast	*Debaryomyces* sp.	N	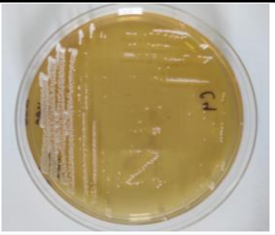	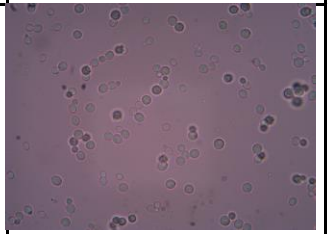
4	C	C23	498.6	IND	*Rachycentron canadum*	FFP	27	35	0.25	23	**C10**	round	yeast	*Debaryomyces* sp.	M	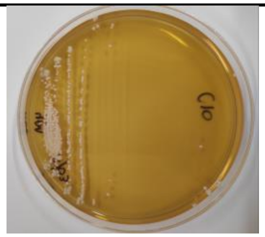	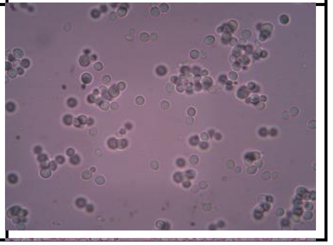

5	C	C27	366.8	IND	*Rachycentron canadum*	FF	27	35	0.73	27	**C12**	round	yeast	*Debaryomyces* sp.	M	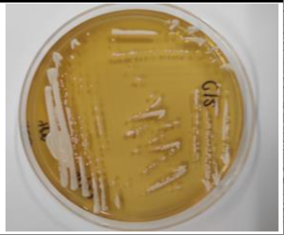	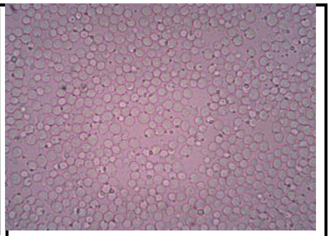
6	C	C32	5200.0	female	*Rachycentron canadum*	FFP	27	35	0.29	32	**C13**	oval	yeast	*Candida haemuloni*	B	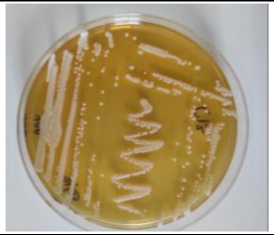	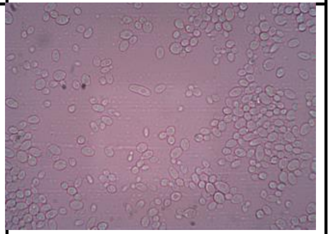
7	C	C32	5200.0	female	*Rachycentron canadum*	FFP	27	35	0.29	32	**C16**	round	yeast	*Debaryomyces* sp.	L	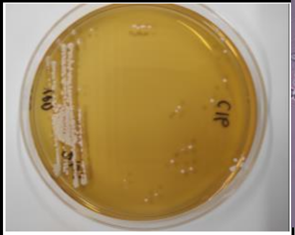	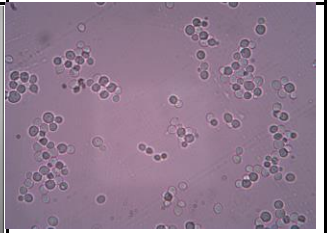
8	C	C32	5200.0	female	*Rachycentron canadum*	FFP	27	35	0.29	32	**C17**	round	yeast	*Debaryomyces* sp.	L	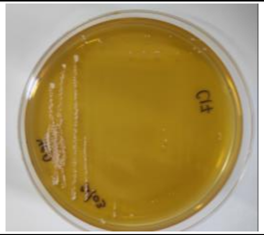	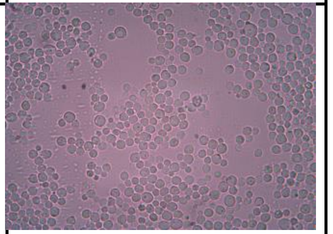
9	C	C28	370.6	IND	*Rachycentron canadum*	FF	27	35	0.59	28	**C19**	round big	yeast	*Debaryomyces* sp.	J	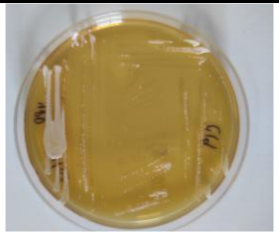	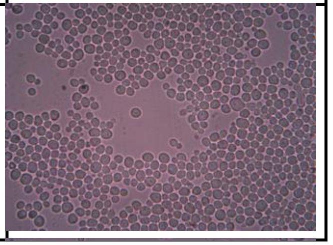

10	C	C24	489.2	IND	*Rachycentron canadum*	FFP	27	35	0.17	24	**C21**	round	yeast	*Debaryomyces* sp.	K	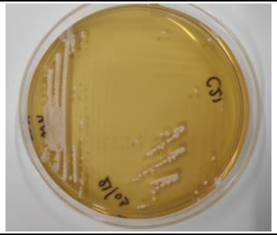	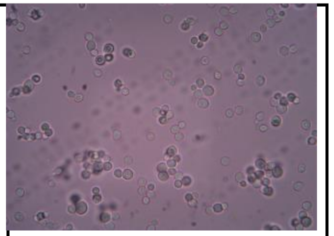
11	C	C27	366.8	IND	*Rachycentron canadum*	FF	27	35	0.73	27	**C22**	oval	yeast	*Candida haemuloni*	B	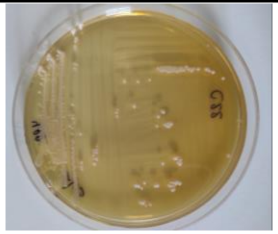	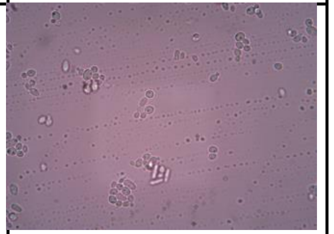
12	E	C5	871.8	female	*Rachycentron canadum*	FFP	26.6	29	0.22	5.2	**C24**	round	yeast	*Debaryomyces* sp.	L	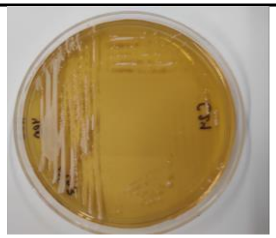	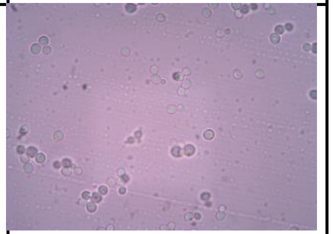
13	E	C1	801.8	male	*Rachycentron canadum*	FFP	26.6	29	0.17	1.2	**C25**	round	yeast	*Debaryomyces* sp.	L	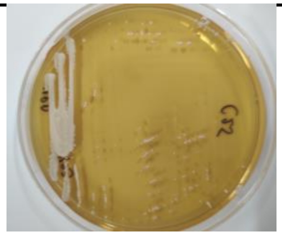	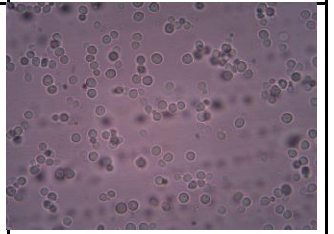
14	E	C1	801.8	male	*Rachycentron canadum*	FFP	26.6	29	0.17	1.2	**C26**	round	yeast	*Debaryomyces* sp.	N	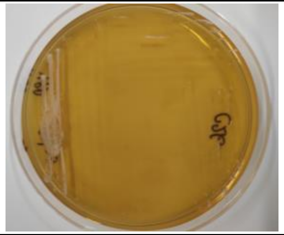	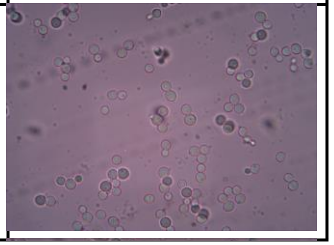

15	E	C5	871.8	female	*Rachycentron canadum*	FFP	26.6	29	0.22	5.2	**C27**	oval	yeast	*Candida haemuloni*	C	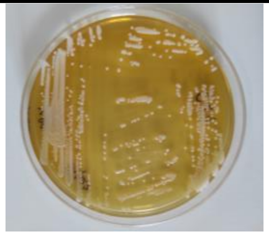	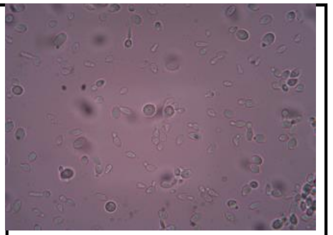
16	E	C5	871.8	female	*Rachycentron canadum*	FFP	26.6	29	0.22	5.2	**C28**	round	yeast	*Debaryomyces* sp.	I	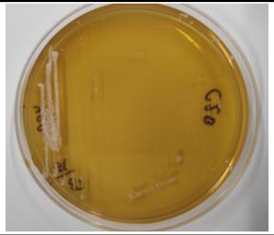	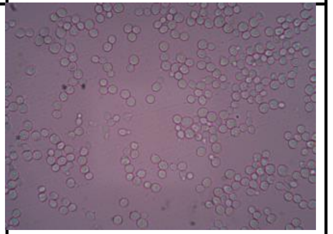
17	E	C2	1447.8	male	*Rachycentron canadum*	FFP	26.6	29	0.21	2.2	**C29**	oval	yeast	*Candida haemuloni*	B	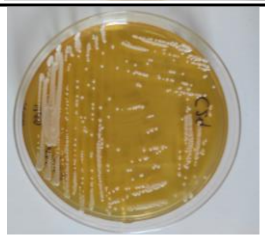	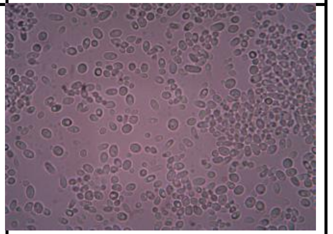
18	E	C1	801.8	male	*Rachycentron canadum*	FFP	26.6	29	0.17	1.2	**C30**	round	yeast	*Debaryomyces* sp.	L	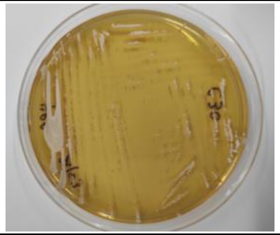	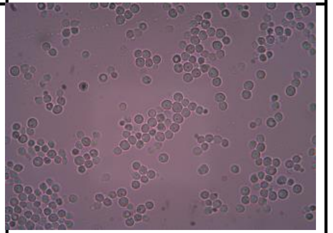
19	E	C2	1447.8	male	*Rachycentron canadum*	FFP	26.6	29	0.21	2.2	**C31**	round big	yeast	*Candida parapsilosis*	F	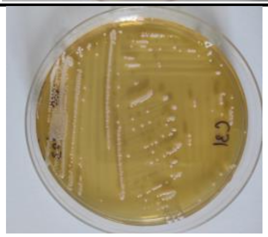	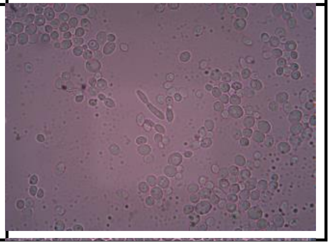

20	E	C6	1271.2	female	*Rachycentron canadum*	FFP	26.6	29	0.32	6.2	**C32**	round big	yeast	*Candida parapsilosis*	G	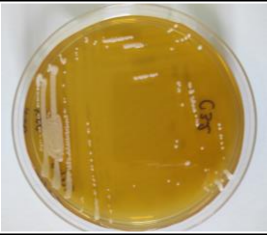	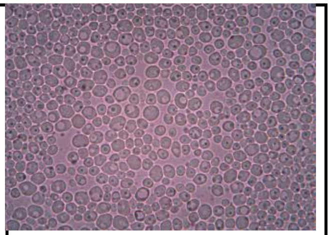
21	E	C1	801.8	male	*Rachycentron canadum*	FFP	26.6	29	0.17	1.2	**C33**	round	yeast	*Candida haemuloni*	D	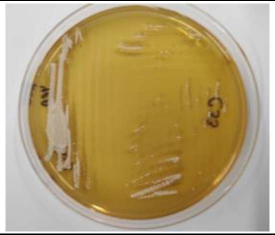	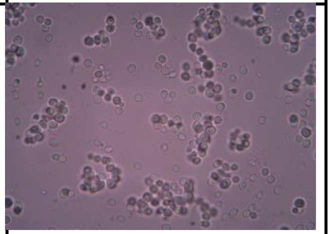
22	E	C6	1271.2	female	*Rachycentron canadum*	FFP	26.6	29	0.32	6.2	**C36**	round big	yeast	*Candida parapsilosis*	H	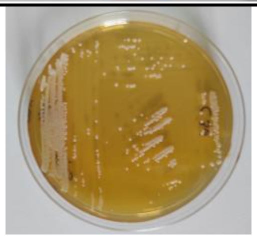	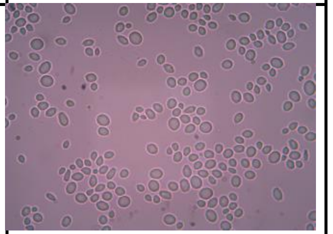
23	E	C1	801.8	male	*Rachycentron canadum*	FFP	26.6	29	0.17	1.2	**C37**	oval	yeast	Candida haemuloni	B	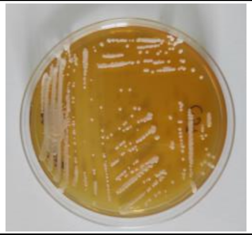	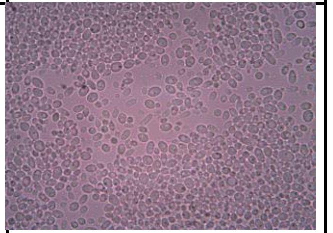
24	E	C1	801.8	male	*Rachycentron canadum*	FFP	26.6	29	0.17	1.2	**C38**	round	yeast	*Debaryomyces* sp.	L	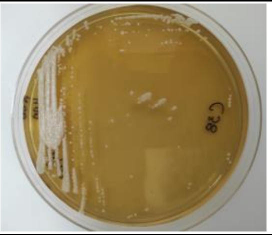	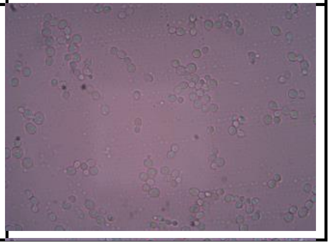

25	C	C20	511.2	IND	*Rachycentron canadum*	FFP	27	35	0.55	20	**C40**	round	yeast	*Debaryomyces* sp.	J	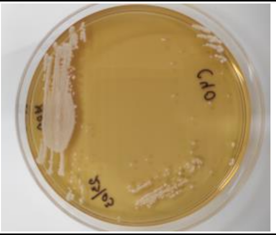	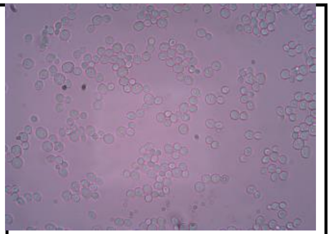
26	C	C20	511.2	IND	*Rachycentron canadum*	FFP	27	35	0.55	20	**C41**	round	yeast	*Debaryomyces* sp.	L	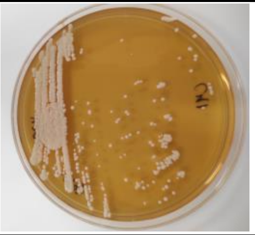	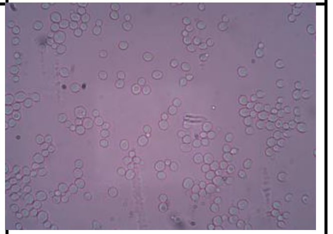
27	C	C27	366.8	IND	*Rachycentron canadum*	FF	27	35	0.73	27	**C42**	oval	yeast	*Candida haemuloni*	B	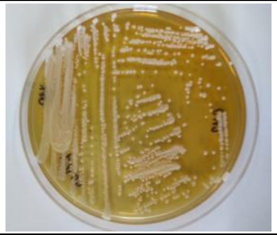	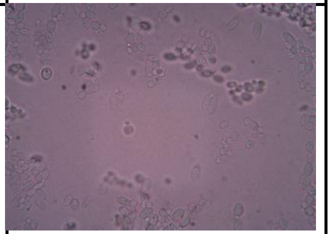
28	C	C25	410.4	IND	*Rachycentron canadum*	FFP	27	35	0.22	25	**C43**	round	yeast	*Debaryomyces* sp.	M	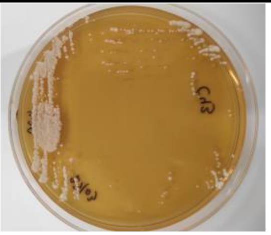	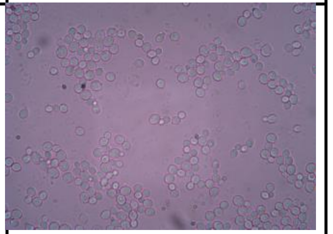
29	C	C23	498.6	IND	*Rachycentron canadum*	FFP	27	35	0.25	23	**C44**	oval	yeast	*Candida haemuloni*	B	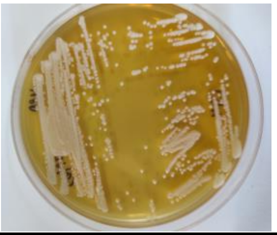	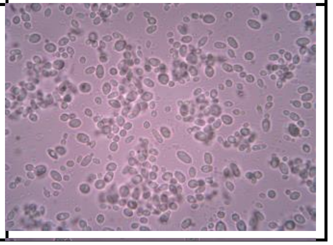

30	C	C23	498.6	IND	*Rachycentron canadum*	FFP	27	35	0.25	23	**C45**	round big	yeast	*Candida haemuloni*	A	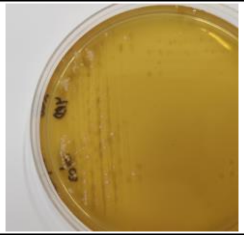	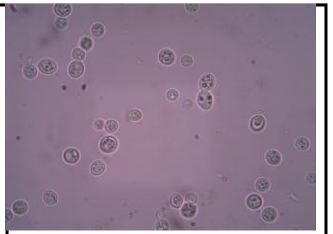
31	E	C6	1271.2	female	*Rachycentron canadum*	FFP	26.6	29	0.32	6.2	**C46**	oval + pseudohypha e	yeast	*Candida parapsilosis*	E	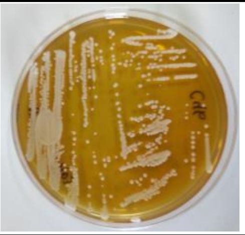	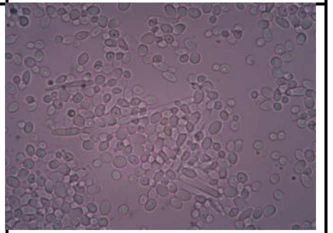
32	E	C4	1410.2	female	*Rachycentron canadum*	FFP	26.6	29	0.4	4.2	**C47**	round big	yeast	*Candida haemuloni*	A	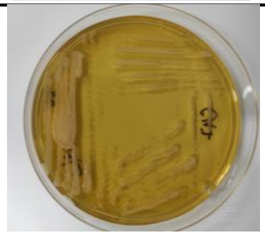	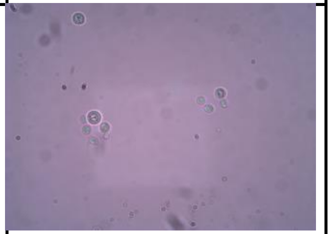
33	C	C30	327.6	IND	*Rachycentron canadum*	FF	27	35	0.31	30	**C49**	round	yeast	*Debaryomyces* sp.	K	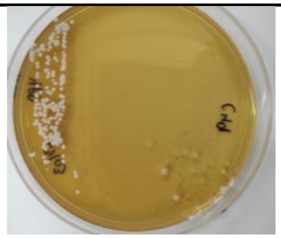	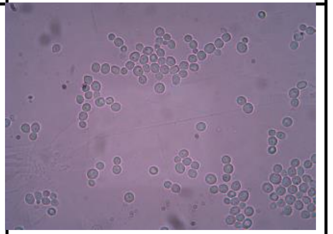
34	E	C6	1271.2	female	*Rachycentron canadum*	FFP	26.6	29	0.32	6.2	**C50**	round	yeast	*Candida parapsilosis*	E	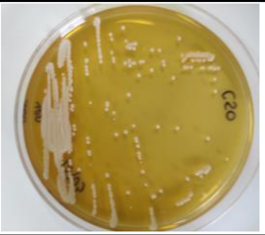	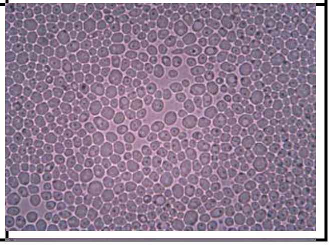

35	C	C27	366.8	IND	*Rachycentron canadum*	FF	27	35	0.73	27	**C60**	round	yeast	*Debaryomyces* sp.	M	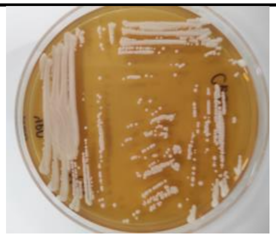	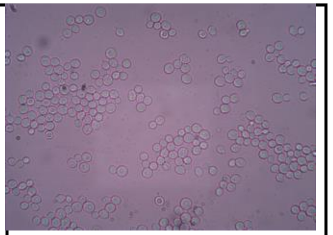
36	E	C1	801.8	male	*Rachycentron canadum*	FFP	26.6	29	0.17	1.2	**C61**	round big	yeast	*Naganishia* sp.	P	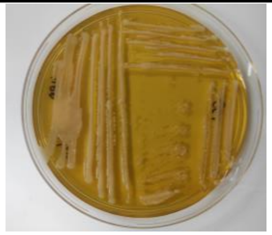	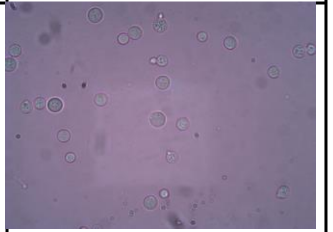
37	E	C3	1087.6	female	*Rachycentron canadum*	FFP	26.6	29	0.24	3.2	**C62**	round	yeast	*Debaryomyces* sp.	K	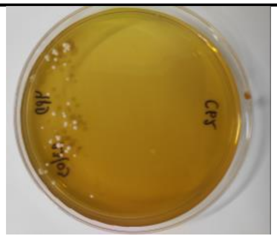	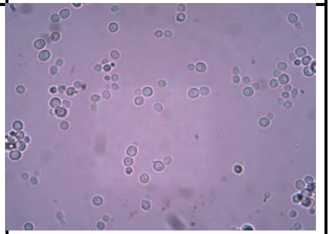
38	C	C25	410.4	IND	*Rachycentron canadum*	FFP	27	35	0.22	25	**C65**	round	yeast	*Debaryomyces* sp.	M	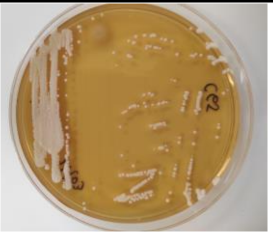	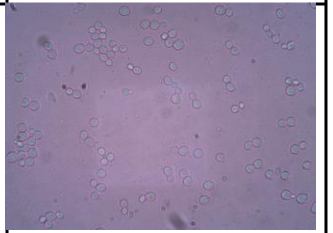
39	E	C1	801.8	male	*Rachycentron canadum*	FFP	26.6	29	0.17	1.2	**C67**	round	yeast	*Debaryomyces* sp.	O	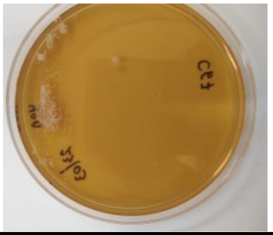	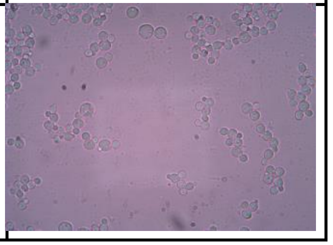

Feed	FF	formulated feed
	FFP	frozen fish pieces

Origin	C	CENAIM
	E	Emagrocom
