# Supplementary material for: Selection of Autochthonous Yeasts Isolated from the Intestinal Tracts of Cobia Fish (Rachycentron canadum) with Probiotic Potential
Source: J Fungi (Basel). 2023 Feb 18;9(2):274. doi: 10.3390/jof9020274 (PMC9966584; doi:10.3390/jof9020274)
Supplement: Supplementary file 1 [file jof-09-00274-s001.zip › Supplementary material/Table S2_rev1.pdf]

Table S2. Results of the second selection step of yeast strains.

N°	Yeast strains	Biomass Production (dry weight)	Biofilm production	Hidrophobicity Xylene	Hidrophobicity Ethyl Acetate	Hidrophobicity Chloroform	Auto-aggregation 1 hour	Auto-aggregation 24 hours
		g/L	Absorbance	%	%	%	%	%
**1**	**Ch-C01**	**2.00 ± 0.07**	**0.91 ± 0.12**	**30.68 ± 0.54**	**25.61 ± 2.26**	**92.15 ± 0.50**	**1.90 ± 0.002**	**81.35 ± 0.006**
**2**	**Ch-C27**	**1.72 ± 0.01**	**1.00 ± 0.21**	**45.22 ± 1.88**	**18.98 ± 4.08**	**88.64 ± 2.48**	**13.85 ± 0.002**	**85.55 ± 0.011**
3	Ch-C47	0.69 ± 0.13	0.63 ± 0.01	53.16 ± 2.60	21.81 ± 3.57	74.53 ± 2.68	22.05 ± 0.002	84.60 ± 0.007
**4**	**Cp-C31**	**1.50 ± 0.04**	**1.44 ± 0.02**	**55.05 ± 1.32**	**49.46 ± 2.57**	**90.57 ± 0.93**	**21.75 ± 0.004**	**87.50 ± 0.006**
**5**	**Cp-C32**	**1.36 ± 0.07**	**1.37 ± 0.07**	**72.77 ± 4.71**	**44.66 ± 0.48**	**94.39 ± 0.66**	**5.05 ± 0.003**	**89.70 ± 0.003**
6	Cp-C33	0.83 ± 0.09	0.83 ± 0.03	72.25 ± 1.28	40.49 ± 5.09	93.21 ± 0.65	25.70 ± 0.004	82.35 ± 0.006
7	Cp-C36	1.59 ± 0.04	1.49 ± 0.07	16.24 ± 4.09	44.27 ± 1.54	92.85 ± 0.77	20.70 ± 0.003	87.55 ± 0.012
**8**	**Cp-C46**	**1.57 ± 0.02**	**1.35 ± 0.03**	**18.56 ± 3.81**	**49.41 ± 2.19**	**93.50 ± 0.29**	**48.15 ± 0.003**	**87.85 ± 0.006**
9	Dh-C03	1.00 ± 0.15	0.26 ± 0.03	43.48 ± 3.82	60.26 ± 4.63	88.45 ± 2.26	17.30 ± 0.002	92.15 ± 0.007
**10**	**Dh-C10**	**1.02 ± 0.08**	**0.13 ± 0.02**	**41.47 ± 3.57**	**59.76 ± 1.27**	**85.25 ± 1.75**	**27.65 ± 0.001**	**89.85 ± 0.005**
11	Dh-C17	0.89 ± 0.04	0.14 ± 0.01	31.40 ± 2.08	36.96 ± 2.96	92.72 ± 1.06	14.00 ± 0.006	84.05 ± 0.006
**12**	**Dh-C28**	**1.05 ± 0.06**	**0.14 ± 0.02**	**43.52 ± 1.56**	**36.07 ± 2.31**	**90.94 ± 0.90**	**6.90 ± 0.001**	**85.30 ± 0.001**
13	Dh-C40	0.93 ± 0.09	0.69 ± 0.03	78.20 ± 2.21	35.16 ± 0.93	88.96 ± 0.22	31.00 ± 0.006	87.20 ± 0.003
14	Dsp-C26	0.87 ± 0.06	0.12 ± 0.00	57.59 ± 1.01	34.85 ± 5.10	82.24 ± 3.60	10.80 ± 0.002	84.70 ± 0.008
15	Dsp-C67	0.30 ± 0.07	0.85 ± 0.03	77.68 ± 0.78	50.38 ± 2.65	93.40 ± 0.19	10.70 ± 0.003	80.25 ± 0.003
16	Nsp-C61	0.66 ± 0.02	0.12 ± 0.00	57.62 ± 2.80	33.99 ± 3.31	90.54 ± 0.12	37.40 ± 0.003	78.00 ± 0.006

data: mean ± standar error

data in bold correspond to the 7 selected yeasts.
