# Supplementary material for: Selection of Autochthonous Yeasts Isolated from the Intestinal Tracts of Cobia Fish (Rachycentron canadum) with Probiotic Potential
Source: J Fungi (Basel). 2023 Feb 18;9(2):274. doi: 10.3390/jof9020274 (PMC9966584; doi:10.3390/jof9020274)
Supplement: Supplementary file 1 [file jof-09-00274-s001.zip › Supplementary material/Table S3_rev1.pdf]

Table S3. Polyamine quantification in the cell pellets and supernatants of the 7 yeast strains.

			Spermidine		Spermine		Total polyamines	
N°	**Yeast strains**	**Origin**	**mean ± se**	**HSD**	**mean ± se**	**HSD**	**mean ± se**	**HSD**
1	Ch-C1	Cell pellet	120.96 ± 16.27	ab	136.69 ± 11.20		257.65 ± 27.46	
2	Ch-C27	Cell pellet	100.33 ± 10.75	b	138.89 ± 17.42		239.22 ± 28.16	
3	Cp-C31	Cell pellet	217.79 ± 24.73	a	175.91 ± 11.44		393.70 ± 36.14	
4	Cp-C32	Cell pellet	209.19 ± 26.91	a	172.91 ± 23.83		382.11 ± 50.44	
5	Cp-C46	Cell pellet	191.57 ± 27.21	ab	149.80 ± 19.56		341.38 ± 45.50	
6	Dh-C10	Cell pellet	147.43 ± 16.80	ab	184.88 ± 7.81		332.31 ± 24.52	
7	Dh-C28	Cell pellet	203.69 ± 11.93	a	195.54 ± 7.46		399.23 ± 19.26	
1	Ch-C1	Supernatant	13.74 ± 0.87	bc	84.22 ± 3.45	ab	97.96 ± 4.21	ab
2	Ch-C27	Supernatant	15.97 ± 2.25	abc	94.51 ± 12.89	ab	110.48 ± 15.03	ab
3	Cp-C31	Supernatant	13.11 ± 1.05	bc	103.26 ± 7.49	ab	116.38 ± 8.54	ab
4	Cp-C32	Supernatant	12.98 ± 1.62	bc	88.68 ± 11.05	ab	101.66 ± 12.61	ab
5	Cp-C46	Supernatant	11.92 ± 1.16	c	84.67 ± 11.43	b	96.59 ± 12.58	b
6	Dh-C10	Supernatant	27.00 ± 2.49	a	165.55 ± 5.31	a	192.55 ± 7.44	a
7	Dh-C28	Supernatant	20.70 ± 0.70	ab	160.11 ± 5.20	a	180.81 ± 5.88	a

data: **mean ± standar error**
HSD: **Honestly-significant-difference Tukey**
