# Supplementary material for: Selection of Autochthonous Yeasts Isolated from the Intestinal Tracts of Cobia Fish (Rachycentron canadum) with Probiotic Potential
Source: J Fungi (Basel). 2023 Feb 18;9(2):274. doi: 10.3390/jof9020274 (PMC9966584; doi:10.3390/jof9020274)
Supplement: Supplementary file 1 [file jof-09-00274-s001.zip › Supplementary material/Table S4_rev1.pdf]

Table S4. Survival of cobia larvae in safety and protection experiments.

	**Safety (24 h)**	**Protective action against a hyposaline stress (8 h)**
**Yeast strain**	**mean ± se**	**mean ± se**
Ch-C01	16.67 ± 2.32 b	33.33 ± 3.33 a
**Dh-C10**	**54.00 ± 7.21 bc**	**46.67 ± 3.33 b**
**Ch-C27**	**35.33 ± 2.67 abc**	**56.67 ± 3.33 a**
**Dh-C28**	**49.33 ± 4.81 bc**	**56.67 ± 3.33 c**
Cp-C31	18.00 ± 4.00 c	0.00 ± 0.00 a
Cp-C32	12.00 ± 6.00 bc	53.33 ± 6.67 b
Cp-C46	18.00 ± 1.15 a	33.33 ± 6.67 ab
Ccontrol	34.00 ± 5.03 ab	53.33 ± 6.67 a

data: mean ± standar error
test: Dunett test
data in bold correspond to the selected yeast strains
